# Synthesis and Antiproliferative Activity of Steroidal Diaryl Ethers

**DOI:** 10.3390/molecules28031196

**Published:** 2023-01-25

**Authors:** Édua Kovács, Hazhmat Ali, Renáta Minorics, Péter Traj, Vivien Resch, Gábor Paragi, Bella Bruszel, István Zupkó, Erzsébet Mernyák

**Affiliations:** 1 Department of Organic Chemistry, University of Szeged, H-6720 Szeged, Hungary; 2 Institute of Pharmacodynamics and Biopharmacy, University of Szeged, H-6720 Szeged, Hungary; 3 Department of Medicinal Chemistry, University of Szeged, H-6720 Szeged, Hungary; 4 Institute of Physics, University of Pécs, H-7625 Pécs, Hungary; 5 Department of Theoretical Physics, University of Szeged, H-6720 Szeged, Hungary

**Keywords:** Chan–Lam reaction, diaryl ether, 13α-estrone, antiproliferative effect, tubulin polymerization, molecular dynamics

## Abstract

Novel 13α-estrone derivatives have been synthesized via direct arylation of the phenolic hydroxy function. Chan–Lam couplings of arylboronic acids with 13α-estrone as a nucleophilic partner were carried out under copper catalysis. The antiproliferative activities of the newly synthesized diaryl ethers against a panel of human cancer cell lines (A2780, MCF-7, MDA-MB 231, HeLa, SiHa) were investigated by means of MTT assays. The quinoline derivative displayed substantial antiproliferative activity against MCF-7 and HeLa cell lines with low micromolar IC_50_ values. Disturbance of tubulin polymerization has been confirmed by microplate-based photometric assay. Computational calculations reveal significant interactions of the quinoline derivative with the taxoid binding site of tubulin.

## 1. Introduction

**Figure 1:**
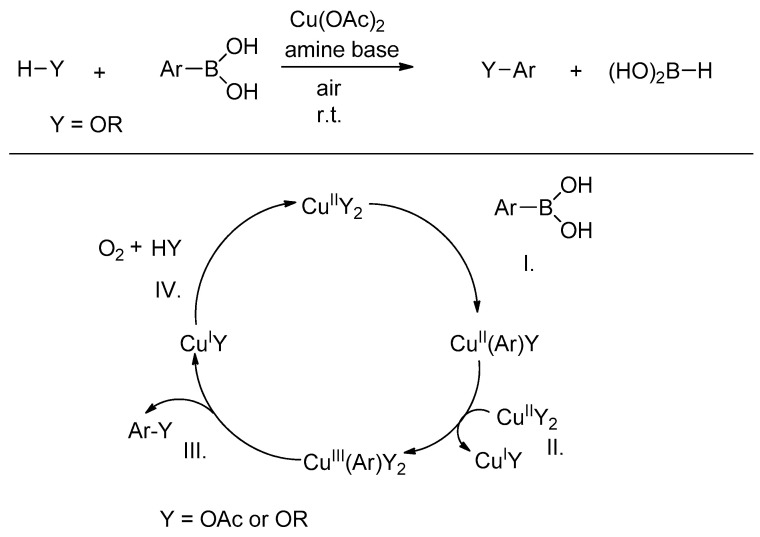
Chan–Lam coupling: C–O bond formation and its proposed mechanism [13].

The development of highly efficient, environmentally friendly catalytic synthetic methods is one of the major goals of modern organic chemistry [1]. Carbon–heteroatom (C–X) bond formation is often a key challenge for organic chemists [2,3,4,5,6,7,8]. It is still desirable to develop mild but effective methods, which allow the construction of the C–X bond with high functional group tolerance. The Ullmann reaction is a traditional method using aryl halides and nucleophiles under transition metal catalysis [9,10]. Nevertheless, owing to its harsh reaction conditions, it is not applicable in certain cases. At the turn of the Millennium, Chan, Evans and Lam published the coupling reactions of arylboronic acids with nucleophiles under the copper salt catalysis, which were later called Chan–Lam coupling reactions [11,12,13]. These transformations are characterized by mild reaction conditions, low toxicity and appropriate stability. The extensions of Chan–Lam couplings allow the establishment of various C–X bonds. The proposed mechanism of the C–O coupling is depicted in Figure 1. After the coordination and transmetalation step (I.), disproportionation (II.) between a CuY_2_ and a Cu^II^(Ar)Y occurs. A reductive elimination (III.) step provides the C–O coupled product. The terminal oxidant is responsible for the oxidation of Cu(I) salts. Application of such methodologies in selective transformations of biologically active compounds might allow feasible syntheses of drug candidates.

**Figure 2:**
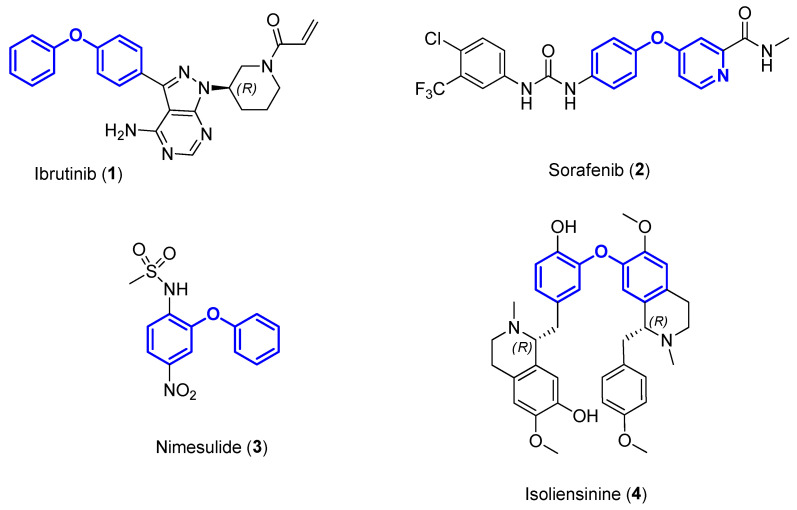
Structures of synthetic or natural DE-based drugs **1**–**4** (the diphenyl ether moiety is highlighted in blue).

The literature describes certain copper-catalyzed methodologies for the synthesis of diphenyl ethers (DEs) [14]. The copper(II)-promoted coupling might even efficiently be carried out at room temperature using an organic or inorganic base in varied solvents. DE represents a compound group bearing two aromatic rings connected via a flexible oxygen bridge. The latter is an essential pharmacophore owing to its substantial hydrophobicity, good lipid solubility, cell membrane penetration and metabolic stability [15,16]. Both synthetic pharmaceuticals [17,18,19,20] and several biologically active natural products [21,22,23,24] contain the DE subunit. DEs, among others, possess anticancer [18,19,20,21], antiviral [25,26], anti-inflammatory [23], antibacterial [27], antiparasitic [28], fungicidal [29], herbicidal [30] or insecticidal [29] activities. Furthermore, the literature reports new efficient DE-based compounds for the therapeutic applications of devastating diseases affecting the central nervous or the cardiovascular system worldwide [14]. Figure 2 shows certain pharmacologically important DE-containing drugs, pesticides and natural products (**1**–**4**). Ibrutinib [17] and sorafenib [18] as small-molecule inhibitors of certain kinase enzymes belong to anti-cancer agents; however, nimesulide [20] is a nonsteroidal anti-inflammatory drug. Isoliensinine [21] displays a variety of biological activities, including anti-cancer, antioxidant and anti-HIV effects.

**Figure 3:**
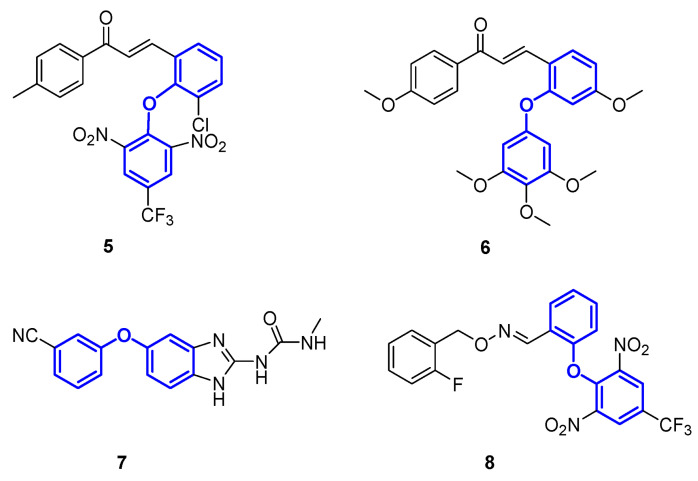
Structures of selected antitubulin agents (**5**–**8**) bearing diphenyl ether moieties highlighted in blue.

Cancer still remains the leading cause of death around the world. The development of new, highly effective anticancer agents with high selectivity is still one of the major goals of medicinal chemistry. Antitubulin compounds are considered to be the most effective tools for cancer chemotherapy nowadays [31]. Certain antitubulin agents have already been approved by the Food and Drug Administration (FDA) [32], but their efficacy might be limited by the development of multidrug-resistant (MDR) cancer cells. Due to their crucial role in cell division, α- and β-tubulin are essential targets for the development of novel anticancer agents. Suppression of cell growth might be achieved by drugs that stabilize or destabilize microtubules (MTs) [33,34]. MT destabilizing agents (MDAs) prevent the polymerization of tubulin and they promote depolymerization, whereas MT stabilizing agents (MSAs) promote the polymerization of tubulin and they stabilize the polymer, preventing depolymerization. Six binding sites have been identified on tubulin [35,36]; however, MSAs generally bind reversibly to the taxoid binding site. Antimitotic drugs approved by FDA might be classified based on their binding site or their further modification (encapsulation or conjugation strategies) [32]. Vincristine sulfate (Oncovin), vinblastine sulfate (Velban) and vinorelbine (Navelbine) belong to the vinca alkaloid site binding drugs; however, colchicine binds to the site with the same name. Marquibo, as a vincristine sulfate liposome injection was developed to improve the pharmacodynamic properties of vincristine. T-DM1 is a maytansine derivative conjugated to trastuzumab applied in a second line breast cancer therapy. It can be stated that antitubulin agents bear a characteristic structure generally. It has two aryl rings and an ethylene, triazole or oxygen bridge, which determine the relative orientation of the rings [36,37]. The latter structural element, the diaryl ether scaffold, is present in certain potent antitubulin agents [15] including substituted or condensed variations (compounds **5**–**8**, Figure 3). Nevertheless, the development of antitubulin compounds possessing improved potency and selectivity is still a leading challenge.

**Figure 4:**
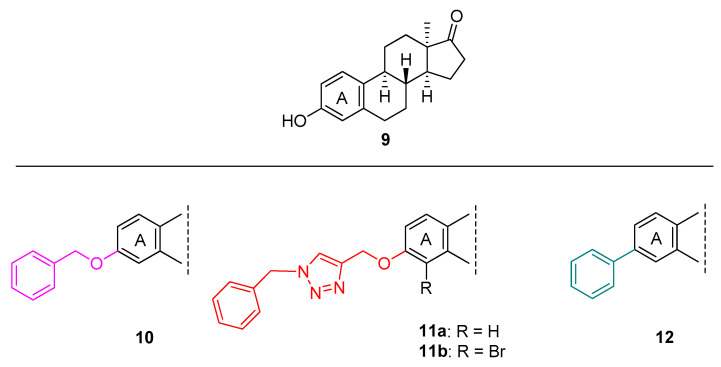
13α-Estrone derivatives **9**–**12** synthesized recently (the modifications are indicated by different colours).

We recently published the syntheses and biochemical investigation of certain biologically active derivatives of the core-modified synthetic 13α-estrone **9**. Inversion of the C-13 in natural estrone derivatives substantially reduces their estrogenic activity [38,39,40,41]. The group of 13α-estrone derivatives proved to be promising concerning their enzyme inhibitory and/or antiproliferative properties. However, their biological activity greatly depends on their structure [33,42,43,44,45,46,47,48]. We have observed that the substitution pattern of ring A influences bioactivity markedly. The introduction of an apolar benzyl group onto the phenolic hydroxy function usually improves the cell growth-inhibitory action of 17-keto or 17-hydroxy 13α-estrone derivatives [42]. Accordingly, the presence of the apolar ether moiety at C-3 seemed to be advantageous. The low micromolar antiproliferative action of benzyl ethers could further be enhanced by inserting a polar triazole ring between the 3-OH and the benzylic moiety [42,48]. Compound **11a** displayed submicromolar IC_50_ values on HeLa, A2780, A431 and MCF-7 cancer cell lines. Introduction of a bromo substituent to the C-4 *ortho* position led to a compound (**11b**) with improved tumor selectivity, being most potent on A2780 cell line (Figure 4) [48]. Mechanistic investigations suggest that compound **11b** exerts a direct effect on microtubule formation. Molecular dynamics (MD, MMGBSA methods) were performed in order to calculate binding energy at an advanced level. Computational calculations revealed strong interactions of compound **11b** with both colchicine (CBS) and taxoid binding sites (TBS) of tubulin with a stronger interaction at the TBS. Consequently, triazole derivative **11b** might be considered as an MSA agent with remarkable tumor selectivity concerning the cell lines investigated. More recently we have shown that 3-deoxy-3-phenyl-13α-estra-1,3,5(10)-triene (**12**) displays weak antiproliferative action [47]. This observation suggests that direct phenylation at C-3 by the simultaneous removal of the hydroxy group is detrimental regarding the antiproliferative action. It follows that the oxygen-containing moiety at C-3 should be retained and its etherification with apolar benzyl or more polar triazolylbenzyl moieties might be highly beneficial. Our results indicate that the hormonally inactive 13α-estrane core with certain ring A modifications might be a suitable scaffold in the design of potent MT targeting agents.

With these considerations in mind, here we aimed to perform the direct arylation of 13α-estrone at C-3-*O* via Chan–Lam coupling using arylboronic acids as reagents. The set of boronic acid coupling partners included not only substituted phenyl but heteroaryl derivatives too. The evaluation of antiproliferative action of the newly synthesized compounds against five human cancer cell lines was also planned. Mechanistic investigations concerning the direct effect of the most potent compound on microtubule formation were also intended. To gain insight into the interaction of the selected potent compound with the taxoid binding site of tubulin, computational calculations were performed.

## 2. Results and Discussion

### 2.1. Chemistry

**Scheme 1:**
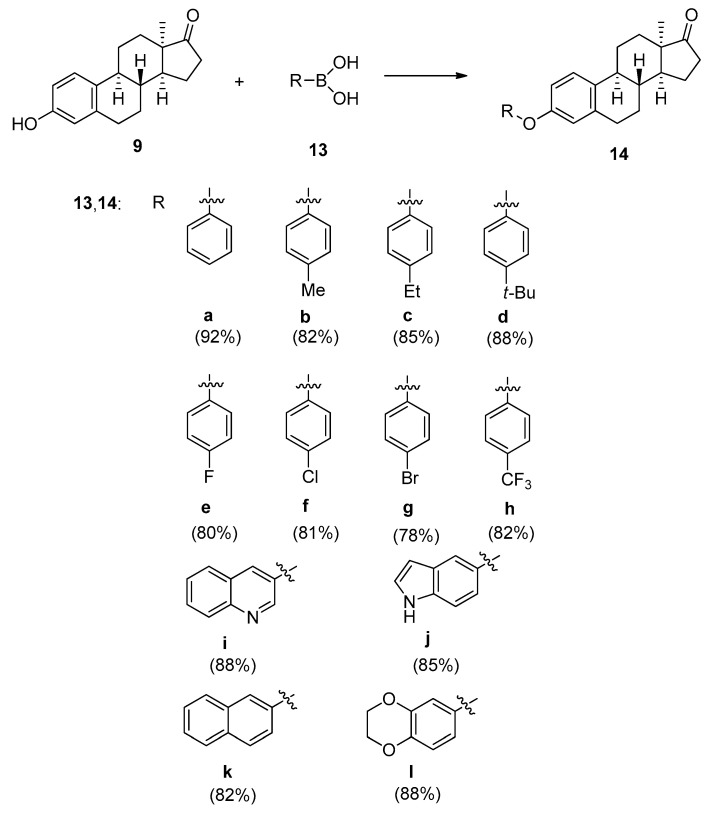
Chan–Lam couplings of 13α-estrone (**9**) with arylboronic acids (**13a**–**l**).

First, the etherification of 13α-estrone **9** with phenylboronic acid **13a** was carried out. Arylboronic acids are outstanding coupling partners in several metal-catalyzed reactions, owing to their broad availability, low toxicity, high stability and extensive functional group tolerance. Based on literature data [12], a copper-promoted C(sp^2^)–O coupling was performed, using 1 equiv. of Cu(OAc)_2_ as the catalyst in dichloromethane solvent. K_2_CO_3_, trimethylamine (NEt_3_) and diisopropylethylamine (DIPEA) were tested as bases but NEt_3_ proved to be the best option based on yields. The protocol was extended to couplings of different arylboronic acids (**13a**–**l**) with 13α-estrone (**9**) (Scheme 1). In order to get important structure–activity information, substituted phenyl (**13a**–**h**) or condensed carbo or heterocyclic derivatives (**13i**–**l**) were chosen as reagents. C(sp^2^)–O couplings proceeded with high isolated yields (Scheme 1).

The structures of the newly synthesized diaryl ethers (**14a**–**l**) were deduced from ^1^H and ^13^C NMR spectra.

### 2.2. Pharmacology

Here we investigated the in vitro cell growth-inhibitory properties of the newly synthesized diaryl ethers (**14a**–**l**) by the MTT assay [49] on a panel of human adherent cancer cell lines. The panel included different cervical (HeLa and SiHa), breast (MCF-7 and MDA-MB-231) and ovarian (A2780) cancer cell lines [50,51,52,53,54,55,56]. The cervical and breast cancer cell line pairs were selected on the basis of their different HPV or receptorial status. NIH/3T3 mouse fibroblast cell line was used for the determination of cancer selectivity.

We have recently described the antiproliferative properties of 13α-estrone-3-benzyl ether (**10**, Figure 4) and its triazolyl derivatives (**11a**,**b**) against HeLa, MCF-7 and A2780 cell lines [42,48]. The etherified compounds (**10**; **11a**,**b**) inhibited the growth of cells more effectively than their 3-OH counterpart (**9**). In both compound types (**10**; **11a**,**b**), the carbo or the heteroaromatic ring was connected to C-3-*O* via a methylene linker. Here we were interested in the investigation of the antiproliferative properties of 13α-estrone derivatives arylated directly at the C-3-*O* function. Although compound structures **14a** and **10** (IC_50_ > 30 μM, HeLa [42]) differ only in a single methylene group, their antiproliferative action varies substantially (Table 1). Compound **14a** is more active, especially on the HPV-18 positive HeLa cervical cell line, displaying a low micromolar IC_50_ value (5.53 µM, Table 1). In addition, the estrogen receptor positive breast cancer cell line MCF-7 proved to be sensitive to **14a** but the IC_50_ value was twice as high as that mentioned previously. Interestingly, **14a** behaved differently on the pairs of breast and cervical cell lines.

All test compounds seemed to be active against the HeLa cell line with IC_50_ values ranging from 5 to 10 µM with the exception of the 4-fluorophenyl derivative **14e** (Table 1). Substitution of a hydrogen with its fluorine bioisostere usually leads to unique biological activity of the fluorinated derivative. The highest electronegativity and the small van der Waals radius make fluorine derivatives generally more active than their unsubstituted counterparts. Nevertheless, in the case of **14e** the presence of fluorine seems to be disadvantageous. If we analyze the results obtained for *para*-tolyl (**14b**) and *para*-trifluoromethyl (**14h**) compounds, only slight differences appear. Accordingly, the effect of fluorine is more pronounced if it is connected directly to the phenyl group.

Introduction of a condensed bicyclic moiety onto the C-3-*O* site resulted in unique structure–activity results. The naphthyl derivative (**14k**) displayed growth-inhibitory actions similar to its phenyl counterpart (**14a**), except on cell lines MCF-7 and SiHa with SiHa being more sensitive to compound **14k**. The presence of a nitrogen heteroatom in the introduced moiety (compound **14i**) resulted in a significant improvement in the biological action. This compound should be highlighted as the most potent derivative on all cell lines investigated (except for SiHa). In contrast, the benzo-1,4-dioxane derivative (**14l**) seemed to be less potent than **14i** on four cancer cell lines. The ovarian cell line A2780 could not distinguish between the two heterocyclic compounds **14i** and **14l**. The other nitrogen-containing heterocyclic derivative (**14j**) was less effective than **14i**.

It should be emphasized that certain newly synthesized derivatives displayed more pronounced cell growth-inhibitory action than the reference compound cisplatin, especially on the HeLa cancer cell line.

In order to get information on the tumor selectivity of the antiproliferative action, the more promising derivatives were investigated on the mouse fibroblast cell line NIH/3T3. None of the compounds exerted inhibition above 50% even at a higher, 30 µM test concentration. In this regard, the reference agent cisplatin proved to be less selective.

It is particularly promising that slight structural modifications generated significant differences in inhibitory activities. This might be indicative of a cell type-dependent action. The broad toxic character of the test compounds might be excluded. On this basis, the compound group investigated might substantially contribute to lead-finding projects based on estrone derivatives. Nevertheless, the mechanistic investigation of antiproliferative action is necessary. The determination of the mechanism of action was inspired by our recent result [48]. We found earlier that certain 13α-estrone derivatives etherified on their phenolic hydroxy function might affect the microtubule formation. On this basis, here we performed an in vitro tubulin polymerization assay with the most potent compound **14i** in two different concentrations (125 and 250 µM). Paclitaxel, a clinically applied anticancer agent was used as a reference compound.

**Figure 5:**
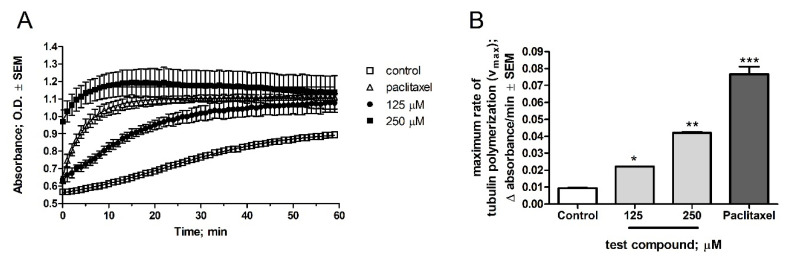
Effects of 14i and 10 μM paclitaxel on the calculated maximum reaction rate (Vmax) of in vitro microtubule formation. (**A**) Representative kinetic curves; (**B**) Calculated results. Control: untreated samples. The experiment was performed in two parallels and the measurements were repeated twice. Each bar denotes the mean ± SEM; *, ** and *** indicate *p* < 0.05, *p* < 0.01 and *p* < 0.001, respectively, compared with the control values.

The obtained absorbance values indicate that **14i** disturbs the polymerization of tubulin by increasing the maximum rate of the procedure. This property was concentration-dependent and statistically significant even at the lower concentration range. The effect of **14i** at 250 μM was lower than that of the reference agent. Based on these in vitro results it can be concluded that the antiproliferative action of **14i** is elicited through the disturbance of tubulin polymerization (Figure 5).

### 2.3. Computational Investigations

Having a picture at the atomic level concerning the possible binding character of the **14i** ligand, MD calculations have been performed. To sample the conformational space of the ligand binding to the taxoid binding site, five independent simulations were run providing a 2.5 μs-long trajectory in total. The resulting protein–ligand interaction diagrams of all five trajectories are presented in Supplementary Materials (Figures S1–S5). According to the simulations, a significant hydrogen-bonding interaction was formed between the ligand and two amino acids (Gln-282 and Thr-276). In one of the trajectories, the Thr-276 amino acid shows extra stabilization with the 17-keto function of the sterane skeleton when the modified steroid finds a deeper position in the taxoid binding site. Both amino acids are located in a flexible loop region near to the taxoid binding pocket and the motion of the loop can affect the binding of the modified steroid. Interestingly, despite that two Gln amino acids located next to each other in position 281 and 282, the ligand had frequent interaction mainly with the second one in the simulations.

**Figure 6:**
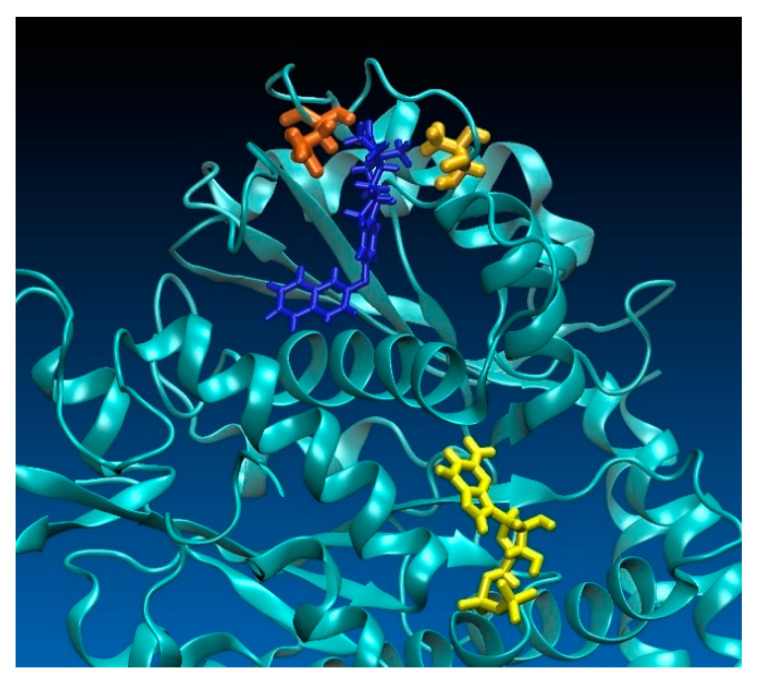
Relative position of compound **14i** (blue), Gln-282 (dark orange), Thr-276 (light orange) and GDP (yellow) nearby the taxoid binding site in the tubulin protein.

Finally, demonstrating the relative position of those amino acids, which had significant interaction with the protein or the guanosine diphosphate (GDP) co-factor, we present a representative ligand–protein complex in Figure 6.

It is worth to mention that the above-mentioned special hydrogen bonds with Gln-282 and Thr-276 always formed between one of the oxygen atoms of the ligand and never with the nitrogen of the quinoline skeleton. Concerning other secondary interactions of the ligand, the analysis showed two further connection types, namely water bridges and hydrophobic interactions. Interestingly, the nitrogen atom in the quinoline ring does not show any specific binding to the tubulin protein.

Finally, we would like to point out that the ligand remained in the binder pocket all along the trajectories, while the GDP left its binding pocket in a number of cases.

## 3. Materials and Methods

### 3.1. Chemistry

Melting points (Mp) were determined with a Kofler hot-stage apparatus. Perkin-Elmer CHN analyzer 2400 was used for the elemental analyses. Thin-layer chromatography was performed on silica gel 60 F_254_ (layer thickness 0.2 mm, Merck); eluent (ss): 30% ethyl acetate/70% hexanes. The spots were detected with I_2_ or UV (365 nm) after spraying with 5% phosphomolybdic acid in 50% aqueous phosphoric acid and heating at 100–120 °C for 10 min. Flash chromatography was performed on silica gel 60, 40–63 μm (Merck). ^1^H NMR spectra were recorded in DMSO-*d_6_* or CDCl_3_ solution with a Bruker DRX-500 instrument at 500 MHz. ^13^C NMR spectra were recorded with the same instrument at 125 MHz under the same conditions. Full scan mass spectra of the newly synthesized compounds were acquired in the range of 50 to 1000 *m*/*z* with a Finnigan TSQ-7000 triple quadrupole mass spectrometer (Finnigan-MAT, San Jose, CA, USA) equipped with a Finnigan electrospray ionization source. Analyses were achieved in positive ion mode applying flow injection mass spectrometry with a mobile phase of 50% aqueous acetonitrile containing 0.1 *v*/*v*% formic acid (flow rate: 0.3 mL/min). Five µL aliquot of the samples were loaded into the flow. The ESI capillary was adjusted to 4.5 kV and N_2_ was used as a nebulizer gas.

### 3.2. General Procedure for the Synthesis of 3-aryloxy-13α-estra-1,3,5(10)-triene-17-ones

13α-Estrone **9** (50 mg, 0.185 mmol), Cu(OAc)_2_ (33 mg, 1 equiv.), arylboronic acid (1 eq.) were dissolved in dichloromethane (5 mL) then triethylamine (125 μL, 5 equiv. mmol) was added. The reaction mixture was stirred at rt for 12–24 h, quenched with water (15 mL) and extracted with dichloromethane (3 × 15 mL). The organic phase was dried over anhydrous Na_2_SO_4_, concentrated in vacuum and the resulting residue was purified by column chromatography. Hexanes were used for crystallization if needed.

*3-Phenoxy-13α-estra-1,3,5(10)-triene-17-one* (**14a**). Reaction time: 16 h. The residue was purified by flash chromatography with hexanes/EtOAc = 6:1 (*v*/*v*) as eluent. Compound **14a** was isolated as a white solid (92%). Mp.: 118.5–119.5 °C; R_f_: 0.38; Mr: 346.2; Anal. Calcd. for C_24_H_26_O_2_: C, 83.20; H, 7.56. Found: C, 83.29; H, 7.52. ^1^H NMR (500 MHz, DMSO-*d_6_*) δ ppm: 0.98 (s, 3H, 13-CH_3_); 2.75 (m, 2H, 6-H_2_); 6.69 (d, 1H, *J* = 2.5 Hz, 4-H); 6.75 (dd, 1H, *J* = 8.5 Hz, *J* = 2.6 Hz, 2-H); 6.95 (d, 2H, *J* = 7.7 Hz, 2′- and 6′-H); 7.10 (t, 1H, *J* = 7.7 Hz, 4′-H); 7.28 (d, 1H, *J* = 8.6 Hz, 1-H); 7.36 (d, 2H, *J* = 7.7 Hz, *J* = 2.0 Hz, 3′- and 5′-H). ^13^C NMR (DMSO-*d_6_*) δ ppm: 20.4 (CH_2_); 24.4 (C-18); 27.5 (CH_2_); 27.8(CH_2_); 29.4 (CH_2_); 31.5 (CH_2_); 32.8 (CH_2_); 40.5 (CH); 40.8 (CH); 48.4 (CH); 49.3 (C-13); 116.1 (CH); 118.2 (2C, 2×CH); 118.3 (CH); 122.9 (CH); 127.3 (CH); 129.8 (2C, 2×CH); 134.8 (C-10); 138.6 (C-5); 154.1 (C); 156.9 (C); 220.5 (C=O). MS *m*/*z* (%) 347 (100, [M + H]^+^).

*3-(4-Tolyloxy)-13α-estra-1,3,5(10)-triene-17-one* (**14b**). Reaction time: 20 h. The residue was purified by flash chromatography with hexanes/EtOAc = 6:1 (*v*/*v*) as eluent. Compound **14b** was isolated as a white solid (82%). Mp.: 147.9–148.7 °C; R_f_: 0.40; M_r_: 360.2; Anal. Calcd. for C_25_H_28_O_2_: C, 83.29; H, 7.83. Found: C, 83.38; H, 7.79. ^1^H NMR (500 MHz, DMSO-*d_6_*) δ ppm: 0.97 (s, 3H, 13-CH_3_); 2.27 (s, 3H, 4′-CH_3_); 2.73 (m, 2H, 6-H_2_); 6.63 (d, 1H, *J* = 2.5 Hz, 4-H); 6.70 (dd, 1H, *J* = 8.5 Hz, *J* = 2.5 Hz, 2-H); 6.85 and 7.15 (2×d, 2×2H, *J* = 8.5 Hz, 2′-, 3′-, 5′- and 6′-H); 7.24 (d, 1H, *J* = 8.6 Hz, 1-H). ^13^C NMR (DMSO-*d_6_*) δ ppm: 20.1 (CH_3_); 20.4 (CH_2_); 24.4 (C-18); 27.5 (CH_2_); 27.8 (CH_2_); 29.4 (CH_2_); 31.5 (CH_2_); 32.8 (CH_2_); 40.5 (CH); 40.7 (CH); 48.4 (CH); 49.3 (C-13); 115.6 (CH); 117.7 (CH); 118.5 (2C, 2×CH); 127.2 (CH); 130.2 (2C, 2×CH); 132.1 (C); 134.3 (C); 138.4 (C); 154.4 (C); 154.7 (C); 220.5 (C=O). MS *m*/*z* (%) 361 (100, [M + H]^+^).

*3-(4-Ethylphenoxy)-13α-estra-1,3,5(10)-triene-17-one* (**14c**). Reaction time: 20 h. The residue was purified by flash chromatography with hexanes/EtOAc = 6:1 (*v*/*v*) as eluent. Compound **14c** was isolated as a white solid (85%). Mp.: 131.7–132.7 °C; R_f_: 0.43; M_r_: 374.2; Anal. Calcd. for C_26_H_30_O_2_: C, 83.38; H, 8.07. Found: C, 83.45; H, 8.01. ^1^H NMR (500 MHz, DMSO-*d_6_*) δ ppm: 0.99 (s, 3H, 13-CH_3_); 1.18 (t, 3H, *J* = 7.6 Hz, -CH_2_-CH_3_); 2.58 (q, 2H, *J* = 7.6 Hz, -CH_2_-CH_3_); 2.75 (m, 2H, 6-H_2_); 6.66 (d, 1H, *J* = 2.5 Hz, 4-H); 6.72 (dd, 1H, *J* = 8.6 Hz, *J* = 2.5 Hz, 2-H); 6.88 and 7.19 (2×d, 2×2H, *J* = 8.4 Hz, 2′-, 3′-, 5′- and 6′-H); 7.25 (d, 1H, *J* = 8.6 Hz, 1-H). ^13^C NMR (DMSO-*d_6_*) δ ppm: 15.4 (-CH_2_-CH_3_); 20.3 (CH_2_); 24.4 (C-18); 27.2 (CH_2_); 27.4 (CH_2_); 27.7 (CH_2_); 29.3 (CH_2_); 31.4 (CH_2_); 32.7 (CH_2_); 40.4 (CH); 40.7 (CH); 48.4 (CH); 49.2 (C-13); 115.6 (CH); 117.8 (CH); 118.3 (2C, 2×CH); 127.0 (CH); 128.8 (2C, 2×CH); 134.3 (C); 138.4 (2C, 2×C); 154.5 (C); 154.6 (C); 220.3 (C=O). MS *m*/*z* (%) 375 (100, [M + H]^+^).

*3-(4-tert-Butylphenoxy)-13α-estra-1,3,5(10)-triene-17-one* (**14d**). Reaction time: 20 h. The residue was purified by flash chromatography with hexanes/EtOAc = 6:1 (*v*/*v*) as eluent. Compound **14d** was isolated as a white solid (88%). Mp.: 129.8–130.8 °C; R_f_: 0.43; M_r_: 402.3; Anal. Calcd. for C_28_H_34_O_2_: C, 83.54; H, 8.51. Found: C, 83.63; H, 8.45. ^1^H NMR (500 MHz, DMSO-*d_6_*) δ ppm: 0.98 (s, 3H, 13-CH_3_); 1.27 (s, 9H, *t*-Bu-CH_3_); 2.75 (m, 2H, 6-H_2_); 6.68 (d, 1H, *J* = 2.5 Hz, 4-H); 6.71 (dd, 1H, *J* = 8.5 Hz, *J* = 2.5 Hz, 2-H); 6.88 and 7.36 (2×d, 2×2H, *J* = 8.4 Hz, 2′-, 3′-, 5′- and 6′-H); 7.25 (d, 2H, *J* = 8.5 Hz, 1-H). ^13^C NMR (DMSO-*d_6_*) δ ppm: 20.3 (CH_2_); 24.4 (C-18); 27.4 (CH_2_); 27.7 (CH_2_); 29.3 (CH_2_); 31.1 (C(CH_3_)_3_); 31.4 (CH_2_); 32.7 (CH_2_); 33.8 (C(CH_3_)_3_); 40.4 (CH); 40.7 (CH); 48.4 (CH); 49.2 (C-13); 115.8 (CH); 117.7 (2C, 2×CH); 118.0 (CH); 126.3 (2C, 2×CH); 127.0 (CH); 134.4 (C); 138.4 (C); 145.2 (C); 154.3 (C); 154.4 (C); 220.3 (C=O). MS *m*/*z* (%) 403 (100, [M + H]^+^).

*3-(4-Fluorophenoxy)-13α-estra-1,3,5(10)-triene-17-one* (**14e**). Reaction time: 12 h. The residue was purified by flash chromatography with hexanes/EtOAc = 6:1 (*v*/*v*) as eluent. Compound **14e** was isolated as a white solid (80%). Mp.: 115.3–116.3 °C; R_f_: 0.41; M_r_: 364.2; Anal. Calcd. for C_24_H_25_FO_2_: C, 79.09; H, 6.91. Found: C, 79.16; H, 6.86. ^1^H NMR (500 MHz, DMSO-*d_6_*) δ ppm: 0.98 (s, 3H, 13-CH_3_); 2.75 (m, 2H, 6-H_2_); 6.67 (d, 1H, *J* = 2.5 Hz, 4-H); 6.72 (dd, 1H, *J* = 8.5 Hz, *J* = 2.5 Hz, 2-H); 7.00 (overlapping doublets, 2H, *J* = 8.7 Hz, 2′- and 6′-H); 7.19 (t, 2H, *J* = 8.7 Hz, 3′- and 5′-H); 7.27 (d, 1H, *J* = 8.6 H, 1-Hz). ^13^C NMR (DMSO-*d_6_*) δ ppm: 20.4 (CH_2_); 24.4 (CH_3_); 27.5 (CH_2_); 27.8 (CH_2_); 29.4 (CH_2_); 31.5 (CH_2_); 32.8 (CH_2_); 40.5 (CH); 40.7 (CH); 48.4 (CH); 49.3 (C); 115.6 (CH); 116.2 (CH); 116.4 (CH); 117.8 (CH); 120.1 (CH); 120.2 (CH); 127.3 (CH); 134.7 (C); 138.6 (C); 152.9 (C); 154.6 (C); 158.8 (C); 220.5 (C=O). MS *m*/*z* (%) 365 (100, [M + H]^+^).

*3-(4-Chlorophenoxy)-13α-estra-1,3,5(10)-triene-17-one* (**14f**). Reaction time: 12 h. The residue was purified by flash chromatography with hexanes/EtOAc = 6:1 (*v*/*v*) as eluent. Compound **14f** was isolated as a white solid (81%). Mp.: 120.7–121.7 °C; R_f_: 0.38; M_r_: 380.2; Anal. Calcd. for C_24_H_25_ClO_2_: C, 75.68; H, 6.62. Found: C, 75.77; H, 6.57. ^1^H NMR (500 MHz, DMSO-*d_6_*) δ ppm: 0.98 (s, 3H, 13-CH_3_); 2.76 (m, 2H, 6-H_2_); 6.72 (d, 1H, *J* = 2.5 Hz, 4-H); 6.78 (dd, 1H, *J* = 8.5 Hz, *J* = 2.5 Hz, 2-H); 6.97 (d, 2H, *J* = 8.9 Hz) and 7.39 (d, 2H, *J* = 8.9 Hz): 2′-, 3′-, 5′- and 6′-H; 7.29 (d, 1H, *J* = 8.6 Hz, 1-H). ^13^C NMR (CDCl_3_) δ ppm: 20.4 (CH_2_); 24.4 (C-18); 27.4 (CH_2_); 27.8 (CH_2_); 29.4 (CH_2_); 31.5 (CH_2_); 32.8 (CH_2_); 40.4 (CH); 40.8 (CH); 48.4 (CH); 49.3 (C-13); 116.3 (CH); 118.6 (CH); 119.7 (2C, 2×CH); 126.6 (C-4′); 127.4 (C-1); 129.6 (2C, 2xCH); 135.3 (C-10); 138.8 (C-5); 153.7 (C); 156.0 (C); 220.5 (C=O). MS *m*/*z* (%) 381 (100, [M + H]^+^).

*3-(4-Bromophenoxy)-13α-estra-1,3,5(10)-triene-17-one* (**14g**). Reaction time: 13 h. The residue was purified by flash chromatography with hexanes/EtOAc = 6:1 (*v*/*v*) as eluent. Compound **14g** was isolated as a white solid (78%). Mp.: 121.3–122.3 °C; R_f_: 0.39; M_r_: 424.1; Anal. Calcd. for C_24_H_25_BrO_2_: C, 67.77; H, 5.92. Found: C, 67.85; H, 5.88. ^1^H NMR (500 MHz, DMSO-*d_6_*) δ ppm: 0.98 (s, 3H, 13-CH_3_); 2.76 (m, 2H, 6-H_2_); 6.73 (d, 1H, *J* = 2.5 Hz, 4-H); 6.78 (dd, 1H, *J* = 8.6 Hz, *J* = 2.5 Hz, 2-H); 6.91 (d, 2H, *J* = 8.9 Hz) and 7.51 (d, 2H, *J* = 8.9 Hz): 2′-, 3′-, 5′- and 6′-H; 7.29 (d, 1H, *J* = 8.6 Hz, 1-H). ^13^C NMR (DMSO-*d_6_*) δ ppm: 20.3 (CH_2_); 24.3 (C-18); 27.4 (CH_2_); 27.7 (CH_2_); 29.3 (CH_2_); 31.4 (CH_2_); 32.7 (CH_2_); 40.4 (CH); 40.7 (CH); 48.4 (CH); 49.2 (C-13); 114.3 (C-4′); 116.2 (CH); 118.5 (CH); 120.0 (2C, 2×CH);127.3 (C-1); 132.4 (2C, 2xCH); 135.3 (C-10); 138.7 (C-5); 153.5 (C); 156.4 (C); 220.3 (C=O). MS *m*/*z* (%) 425 (100, [M + H]^+^).

*3-(4-Trifluoromethylphenoxy)-13α-estra-1,3,5(10)-triene-17-one* (**14h**). Reaction time: 14 h. The residue was purified by flash chromatography with hexanes/EtOAc = 6:1 (*v*/*v*) as eluent. Compound **14h** was isolated as a white solid (82%). Mp.: 123.7–124.7 °C; R_f_: 0.37; M_r_: 414.2; Anal. Calcd. for C_25_H_25_F_3_O_2_: C, 72.45; H, 6.08. Found: C, 72.52; H, 6.01. ^1^H NMR (500 MHz, DMSO-*d_6_*) δ ppm: 0.99 (s, 3H, 13-CH_3_); 2.80 (m, 2H, 6-H_2_); 6.82 (d, 1H, *J* = 2.5 Hz, 4-H); 6.86 (dd, 1H, *J* = 8.5 Hz, *J* = 2.5 Hz, 2-H); 7.09 (d, 2H, *J* = 8.7 Hz) and 7.69 (d, 2H, *J* = 8.7 Hz): 2′-, 3′-, 5′- and 6′-H; 7.34 (d, 1H, *J* = 8.5 Hz, 1-H). ^13^C NMR (DMSO-*d_6_*) δ ppm: 20.3 (CH_2_); 24.4 (C-18); 27.4 (CH_2_); 27.7 (CH_2_); 29.3 (CH_2_); 31.4 (CH_2_); 32.7 (CH_2_); 40.4 (CH); 40.7 (CH); 48.4 (CH); 49.2 (C-13); 117.1 (CH); 117.4 (2C, 2xCH); 119.4 (CH); 122.8 (q, *J* = 32.4 Hz, C); 124.1 (q, *J* = 271.4 Hz, C); 127.1 (q, 2C, *J* = 3.7 Hz, 2×CH); 127.4 (CH); 136.1 (C); 139.0 (C); 152.4 (C); 160.5 (C); 220.3 (C=O). MS *m*/*z* (%) 415 (100, [M + H]^+^).

*3-(Quinolinyl-3-oxy)-13α-estra-1,3,5(10)-triene-17-one* (**14i**). Reaction time: 24 h. The residue was purified by flash chromatography with hexanes/EtOAc = 7:1 (*v*/*v*) as eluent. Compound **14i** was isolated as a white solid (88%). Mp.: 153.2–154.2 °C; R_f_: 0.12; M_r_: 397.2; Anal. Calcd. for C_27_H_27_NO_2_: C, 81.58; H, 6.85. Found: C, 81.67; H, 6.79. ^1^H NMR (500 MHz, DMSO-*d_6_*) δ ppm: 1.00 (s, 3H, 13-CH_3_); 2.80 (m, 2H, 6-H_2_); 6.86 (d, 1H, *J* = 2.6 Hz, 4-H); 6.91 (dd, 1H, *J* = 8.6 Hz, *J* = 2.6 Hz, 2-H); 7.35 (d, 1H, *J* = 8.6 Hz, 1-H); 7.57 (t, 1H, *J* = 8.0 Hz); 7.67 (dt, 1H, *J* = 8.0 Hz, *J* = 1.2 Hz); 7.74 (d, 1H, *J* = 2.7 Hz); 7.90 (d, 1H, *J* = 8.0 Hz); 8.01 (d, 1H, *J* = 8.0 Hz); 8.77 (d, 1H, *J* = 2.7 Hz). ^13^C NMR (DMSO-*d_6_*) δ ppm: 20.3 (CH_2_); 24.3 (C-18); 27.4 (CH_2_); 27.7 (CH_2_); 29.3 (CH_2_); 31.4 (CH_2_); 32.7 (CH_2_); 40.4 (CH); 40.7 (CH); 48.4 (CH); 49.2 (C-13); 116.2 (CH); 118.5 (CH); 119.6 (CH); 127.0 (CH); 127.2 (CH); 127.4 (CH); 127.6 (CH); 128.1 (C); 128.4 (CH); 135.6 (C); 138.9 (C); 143.9 (C); 144.6 (CH); 150.6 (C); 153.4 (C); 220.3 (C=O). MS *m*/*z* (%) 398 (100, [M + H]^+^).

*3-(1H-Indol-5-yloxy)-13α-estra-1,3,5(10)-triene-17-one* (**14j**). Reaction time: 24 h. The residue was purified by flash chromatography with hexanes/EtOAc = 7:1 (*v*/*v*) as eluent. Compound **14j** was isolated as a white solid (85%). Mp.: 205.3–206.3 °C; R_f_: 0.09; M_r_: 385.2; Anal. Calcd. for C_26_H_27_NO_2_: C, 81.01; H, 7.06. Found: C, 81.10; H, 7.01. ^1^H NMR (500 MHz, DMSO-*d_6_*) δ ppm: 0.97 (s, 3H, 13-CH_3_); 2.71 (m, 2H, 6-H_2_); 6.36 (s, 1H); 6.57 (d, 1H, *J* = 2.5 Hz); 6.66 (dd, 1H, *J* = 8.6, *J* = 2.6 Hz); 6.78 (dd, 1H, *J* = 8.6 Hz, *J* = 2.6 Hz); 7.13 (d, 1H, *J* = 2.5 Hz); 7.19 (d, 1H, *J* = 8.6 Hz); 7.35 (t, 1H, *J* = 5.4 Hz); 7.37 (d, 1H, *J* = 8.6 Hz); 11.05 (s, 1H, NH). ^13^C NMR (CDCl_3_) δ ppm: 20.3 (CH_2_); 24.4 (C-18); 27.5 (CH_2_); 27.8 (CH_2_); 29.4 (CH_2_); 31.4 (CH_2_); 32.7 (CH_2_); 40.5 (CH); 40.6 (CH); 48.4 (CH); 49.2 (C); 100.9 (CH); 109.8 (CH); 112.1 (CH); 114.1 (CH); 114.5 (CH); 116.5 (CH); 126.3 (CH); 126.8 (CH); 128.0 (C); 132.6 (C); 133.1 (C); 138.0 (C); 149.0 (C); 156.5 (C); 220.3 (C=O). MS *m*/*z* (%) 386 (100, [M + H]^+^).

*3-(Naphthyl-2-oxy)-13α-estra-1,3,5(10)-triene-17-one* (**14k**). Reaction time: 24 h. The residue was purified by flash chromatography with hexanes/EtOAc = 6:1 (*v*/*v*) as eluent. Compound **14k** was isolated as a white solid (82%). Mp.: 167.8–168.8 °C; R_f_: 0.35; M_r_: 396.2; Anal. Calcd. for C_28_H_28_O_2_: C, 84.81; H, 7.12. Found: C, 84.88; H, 7.08. ^1^H NMR (500 MHz, DMSO-*d_6_*) δ ppm: 0.98 (s, 3H, 13-CH_3_); 2.76 (m, 2H, 6-H_2_); 6.76 (d, 1H, *J* = 2.6 Hz, 4-H); 6.82 (dd, 1H, *J* = 8.6 Hz, *J* = 2.6 Hz, 2-H); 7.24 (dd, 1H, *J* = 8.6 Hz, *J* = 2.6 Hz); 7.31 (d, 1H, *J* = 8.6 Hz); 7.33 (d, 1H, *J* = 2.6 Hz); 7.42 (t, 1H, *J* = 7.1 Hz); 7.47 (t, 1H, *J* = 7.1 Hz); 7.79 (d, 1H, *J* = 8.1 Hz); 7.89 (d, 1H, *J* = 8.1 Hz); 7.93 (d, 1H, *J* = 8.9 Hz). ^13^C NMR (DMSO-*d_6_*) δ ppm: 20.4 (CH_2_); 24.4 (C-18); 27.5 (CH_2_); 27.8 (CH_2_); 29.5 (CH_2_); 31.5 (CH_2_); 32.8 (CH_2_); 40.5 (CH); 40.8 (CH); 48.4 (CH); 49.4 (C-13); 113.1 (CH); 116.3 (CH); 118.5 (CH); 119.5 (CH); 124.6 (CH); 126.5 (CH); 126.9 (CH); 127.3 (CH); 127.5 (CH); 129.5 (C); 129.9 (CH); 133.8 (C); 135.0 (C); 138.7 (C); 154.1 (C); 154.8 (C); 220.5 (C=O). MS *m*/*z* (%) 397 (100, [M + H]^+^).

*3-(2,3-Dihydro-benzo*[1,4]*dioxin-6-yloxy)-13α-estra-1,3,5(10)-triene-17-one* (**14l**). Reaction time: 24 h. The residue was purified by flash chromatography with hexanes/EtOAc = 6:1 (*v*/*v*) as eluent. Compound **14l** was isolated as a white solid (88%). Mp.: 177.2–178.0 °C; R_f_: 0.26; M_r_: 404.2; Anal. Calcd. for C_26_H_28_O_4_: C, 77.20; H, 6.98. Found: C, 77.28; H, 6.93. ^1^H NMR (500 MHz, DMSO-*d_6_*) δ ppm: 0.98 (s, 3H, 13-CH_3_); 2.74 (m, 2H, 6-H_2_); 4.22 (m, 4H, 2′- and 3′-H_2_); 6.45 (dd, 1H, *J* = 8.7 Hz, *J* = 2.8 Hz); 6.48 (d, 1H, *J* = 2.7 Hz); 6.63 (d, 1H, *J* = 2.4 Hz); 6.68 (dd, 1H, *J* = 8.5 Hz, *J* = 2.5 Hz); 6.83 (d, 1H, *J* = 8.7 Hz); 7.23 (d, 1H, *J* = 8.6 Hz). ^13^C NMR (DMSO-*d_6_*) δ ppm: 20.3 (CH_2_); 24.4 (C-18); 27.4 (CH_2_); 27.7 (CH_2_); 29.4 (CH_2_); 31.4 (CH_2_); 32.7 (CH_2_); 40.4 (CH); 40.6 (CH); 48.4 (CH); 49.2 (C-13); 63.6 (CH_2_); 64.0 (CH_2_); 107.7 (CH); 111.6 (CH); 115.1 (CH); 117.3 (CH); 117.4 (CH); 126.9 (CH); 134.1 (C); 138.3 (C); 139.4 (C); 143.6 (C); 150.2 (C); 155.0 (C); 220.3 (C=O). MS *m*/*z* (%) 405 (100, [M + H]^+^).

## 4. Determination of Antiproliferative Activities

The antiproliferative activities of the currently presented molecules **14a**–**l** were determined against human adherent cancer cell lines of gynecological origin. MCF-7 and MDA-MB-231 cells were isolated from breast cancers, while A2780 is an ovarian cancer cell line. In addition, Hela and SiHa cells were isolated from cervical cancers containing HPV-18 and HPV-16, respectively. The tumor selectivity of molecules was determined using nonmalignant mouse embryo fibroblast cells (NIH/3T3). All cell lines were obtained from the European Collection of Cell Cultures (ECCAC, Salisbury, UK) except for SiHa (American Tissue Culture Collection, Manassas, VA, USA). Cells were maintained in minimal essential medium (MEM) completed with 10% fetal calf serum, 1% nonessential amino acids, and an antibiotic–antimycotic mixture. All media and supplements were purchased from Lonza Group Ltd., Basel, Switzerland. Near-confluent tumor and fibroblast cells were plated onto a 96-well microplate at 5000 cells/well density.

After overnight preincubation, a 200 μL new medium containing the tested compounds (at 10 or 30 µM) was added. After incubation (72 h, 37 °C, humidified air, 5% CO_2_), the viable cells were determined by the addition of 3-(4,5-dimethylthiazol-2-yl)-2,5-diphenyltetrazolium bromide (MTT) solution (5 mg/mL, 20 μL). The reagent was metabolized by active mitochondrial reductase and precipitated as purple formazan during a 4 h contact period. Then the medium was discarded, and the crystals were solubilized in 100 μL of DMSO during a 60 min period of shaking at 37 °C. Finally, the produced formazan was assayed at 545 nm, using a microplate reader (SPECTROStar Nano, BMG Labtech, Offenburg, Germany), utilizing untreated cells as control [49]. In the case of the compounds eliciting higher than 50% growth inhibition at 30 µM, the assays were repeated with a set of dilutions and IC_50_ values were calculated from the determined data (sigmoidal curve, GraphPad Prism 5.01, GraphPad Software, San Diego, CA, USA). All experiments were performed on two microplates with at least five parallel conditions. Stock solutions of 10 mM were prepared from the investigated items in DMSO and the highest concentration of the solvent in the medium (0.3%) did not elicit any considerable action on cell growth. Cisplatin (Ebewe Pharma GmbH, Unterach, Austria) was included as a reference agent.

## 5. Tubulin Polymerization Assay

The effect of **14i** on tubulin polymerization was determined by means of a commercially available assay kit as described previously [48]. Briefly, 10 μL of a 125 or 250 μM solution of the tested analog was placed on a prewarmed (37 °C), UV-transparent microplate. Paclitaxel and general tubulin buffer were used as positive and negative control, respectively. A total volume of 100 μL of 3.0 mg/mL tubulin in 80 mM PIPES with 2 mM MgCl_2_, 0.5 mM EGTA with 1 mM GTP at pH 6.9, was added to the samples to initiate the polymerization. A 60 min kinetic measurement protocol was used to describe the absorbance of the reaction mixture per minute at 340 nm (SpectoStarNano, BMG Labtech, Ortenberg, Germany). The maximum reaction rate (Vmax: Δabsorbance/min) was determined by calculating the moving averages of absorbances at three consecutive time points. The highest difference between two succeeding moving averages was considered as the Vmax of the tested molecule. Each sample was prepared in two parallels and the measurements were repeated twice. For statistical evaluation, Vmax data were analysed by the one-way ANOVA test with the Newmann–Keuls post-test by using Prism 5.01 software (GraphPad Software, San Diego, CA, USA).

## 6. Computational Simulations

For molecular dynamics (MD) simulations, a taxol-stabilized microtubule complex (PDB entry: 5SYF) was selected [57]. The original PDB structure was prepared for further calculations using the Protein Preparation Wizard module of the Schrodinger Maestro program [58,59]. It consists of the addition of missing hydrogen atoms under physiological pH condition as well as the missing loops or side chains. The prepared pdb structure was relaxed by a short (5 ns) MD simulation and the last frame of the relaxation running was selected as the target protein for the docking calculations. Docking was performed with the Glide package of the Schrödinger suite [60], using the extra-precision docking protocol. To sample the conformational space, 5 independent MD simulations of 500 ns length were run in a cubic box with a 10 Å buffer size and 0.15 M salt concentration by the Desmond package [61]. The initial structure of the production MD run was selected as the outcome of the docking calculation with the best docking score. In all MD runs, the OPLS4 force field and a simple point charge (SPC) water model were applied [62].

## 7. Conclusions

Antitubulin compounds represent the most effective class of anticancer agents. The more we understand about the structure–activity relationship of these compounds, the better options we have in utilizing them in fight against cancer. The literature reveals characteristic structural elements responsible for antitubulin effect, including the diaryl ether scaffold. In order to develop novel potential antitubulin compounds, we synthesized 13α-estrone 3-*O*-aryl derivatives via Chan–Lam coupling reactions. The copper-catalyzed etherification of the steroidal phenolic hydroxy function was achieved using arylboronic acids as coupling partners. Carbo or heterocyclic rings, bearing different substituents were introduced. The antitumoral properties of the newly synthesized 13α-estrone derivatives were investigated in vitro on a panel of human adherent cancer cell lines (A2780, MCF-7, MDA-MB 231, HeLa and SiHa). Certain compounds exerted more pronounced antiproliferative action than the reference agent cisplatin. The HeLa cancer cell line seemed to be the most sensitive to test compounds. The quinoline derivative **14i** should be highlighted as the most potent steroid against MCF-7 and HeLa cell lines. The tumor selectivity of the test compounds proved to be higher than that of the reference agent cisplatin. Compound **14i** might be regarded as a MT stabilizing agent, since it exerted its antiproliferative effect through the disturbance of tubulin polymerization. Significant interactions of the **14i** derivative with the taxoid binding site of tubulin were identified by computational simulations. Our results might contribute to the development of more potent antitubulin agents with high selectivity.

## Figures and Tables

**Table 1:** Antiproliferative activities of newly synthesized diaryl ethers (**14a**–**l**).

Antiproliferative Activities of Newly Synthesized Diaryl Ethers (14a–l)							
Compd.	Conc. (μM)	Inhibition (%) ± SEM (Calculated IC_50_ Value; μM)					
		MCF-7	MDA-MB-231	HeLa	SiHa	A2780	NIH/3T3
**14a**	10	37.52 ± 2.46	– *	60.24 ± 0.58	–	23.00 ± 1.32	-
	30	81.42 ± 1.50 (10.12)	25.30 ± 1.82	92.02 ± 1.38 (5.53)	–	58.48 ± 1.03 (23.81)	38.75 ± 1.21
**14b**	10	–	–	56.69 ± 1.29	–	39.64 ± 1.84	–
	30	29.28 ± 1.26	33.34 ± 1.75	83.29 ± 1.34 (7.99)	–	62.15 ± 1.15 (16.67)	43.34 ± 2.17
**14c**	10	43.99 ± 1.77	–	62.38 ± 1.19	27.24 ± 2.60	–	–
	30	58.64 ± 1.08	33.16 ± 0.95	88.14 ± 0.79	43.55 ± 0.86	45.08 ± 1.75	21.99 ± 2.03
		(16.44)		(5.13)			
**14d**	10	24.75 ± 0.74	-	65.28 ± 1.02	-	30.84 ± 2.24	–
	30	30.77 ± 1.67	32.93 ± 1.88	77.06 ± 0.93 (7.11)	41.14 ± 2.38	56.98 ± 1.07 (23.65)	24.07 ± 1.67
**14e**	10	49.02 ± 0.85	–	30.14 ± 3.05	–	–	–
	30	60.22 ± 1.68 (13.28)	–	37.78 ± 3.86	–	46.50 ± 2.43	–
**14f**	10	-	-	60.72 ± 1.24	–	34.93 ± 2.85	–
	30	37.51 ± 1.44	30.28 ± 2.24	75.40 ± 1.42 (5.78)	38.91 ± 1.69	51.48 ± 1.93 (26.17)	–
**14g**	10	48.82 ± 2.18	–	65.97 ± 0.61	20.31 ± 3.10	–	–
	30	59.91 ± 1.73 (11.98)	28.28 ± 1.39	80.70 ± 0.76 (5.21)	38.42 ± 1.30	39.58 ± 2.60	30.92 ± 2.15
**14h**	10	35.08 ± 0.69	27.06 ± 2.93	67.46 ± 0.46	31.80 ± 2.77	36.13 ± 1.39	–
	30	45.42 ± 3.30	39.67 ± 2.39	80.74 ± 0.58 (6.90)	45.55 ± 2.63	52.60 ± 1.00 (23.47)	45.47 ± 0.28
**14i**	10	49.68 ± 2.46	24.07 ± 2.47	76.18 ± 1.49	22.46 ± 2.36	48.66 ± 1.90	35.48 ± 1.33
	30	74.67 ± 0.51 (8.52)	58.28 ± 1.39 (22.95)	90.11 ± 0.80 (3.98)	48.33 ± 1.27	71.21 ± 1.35 (11.54)	49.87 ± 0.89
**14j**	10	–	–	50.99 ± 2.21	–	–	–
	30	42.25 ± 1.33	–	71.63 ± 1.45 (9.60)	–	28.02 ± 3.57	–
**14k**	10	–	29.61 ± 3.01	73.86 ± 1.48	28.80 ± 2.10	42.23 ± 0.71	–
	30	43.11 ± 2.035	41.87 ± 0.85	77.08 ± 1.34 (5.52)	54.33 ± 0.83 (23.90)	51.25 ± 1.43 (25.25)	24.15 ± 0.60
**14l**	10	29.08 ± 1.88	20.72 ± 1.33	61.30 ± 0.98	20.79 ± 3.34	48.44 ± 0.49	–
	30	63.02 ± 1.09 (18.91)	24.10 ± 3.15	74.01 ± 0.80 (9.16)	38.58 ± 2.61	69.17 ± 0.50 (11.47)	46.16 ± 2.11 –
cisplatin	10	53.03 ± 2.29	20.75 ± 0.81	42.61 ± 2.33	60.98 ± 0.92	83.57 ± 1.21	76.74 ± 1.26
	30	86.90 ± 1.24	74.47 ± 1.20	99.93 ± 0.26	88.95 ± 0.53	95.02 ± 0.28	96.90 ± 0.25
		(5.78)	(19.13)	(12.43)	(4.29)	(1.30)	(4.73)

*: The inhibition value is less than 20% and not given numerically.

## Data Availability

Not applicable.

## Supplementary Materials

The following supporting information can be downloaded at: https://www.mdpi.com/article/10.3390/molecules28031196/s1, Table S1: Antiproliferative activities of compounds **14a**–**l**, Figure S1: Presence of secondary interactions between the protein amino acids and **14i** ligand along the 1st trajectory. Figure S2: Presence of secondary interactions between the protein amino acids and **14i** ligand along the 2nd trajectory. Figure S3: Presence of secondary interactions between the protein amino acids and **14i** ligand along the 3rd trajectory. Figure S4: Presence of secondary interactions between the protein amino acids and **14i** ligand along the 4th trajectory. Figure S5: Presence of secondary interactions between the protein amino acids and **14i** ligand along the 5th trajectory. ^1^H and ^13^C NMR spectra of the newly synthesized compounds.

## Sample Availability

Samples of the compounds are not available from the authors.

## References

[1] Chen J.Q., Li J.H., Dong Z.B. (2020). A Review on the Latest Progress of Chan-Lam Coupling Reaction. Adv. Synth. Catal..

[2] Pal T., Lahiri G.K., Maiti D. (2020). Copper in Efficient Synthesis of Aromatic Heterocycles with Single heteroatom. Eur. J. Org. Chem..

[3] De Nino A., Maiuolo L., Costanzo P., Algieri V., Jiritano A., Olivito F., Tallarida M.A. (2021). Recent Progress in Catalytic Synthesis of 1,2,3-Triazoles. Catalysts.

[4] Yadav P., Bhalla A. (2022). Recent Advances in Green Synthesis of Functionalized Quinolines of Medicinal Impact (2018–Present). ChemistrySelect.

[5] Liu C., Zhang H., Shi W., Lei A.W. (2011). Bond Formations between Two Nucleophiles: Transition Metal Catalyzed Oxidative Cross-Coupling Reactions. Chem. Rev..

[6] Mousseau J.J., Charette A.B. (2013). Direct Functionalization Processes: A Journey from Palladium to Copper to Iron to Nickel to Metal-Free Coupling Reactions. Acc. Chem. Res..

[7] Liang Y., Zhang X., Macmillan D.W.C. (2018). Decarboxylative sp^3^ C–N coupling via dual copper and photoredox catalysis. Nature.

[8] Kong D., Moon P.J., Bsharat O., Lundgren R.J. (2020). Direct Catalytic Decarboxylative Amination of Aryl Acetic Acids. Angew. Chem. Int. Ed..

[9] Zhao H., Yang K., Zhen H.Y., Ding R.C., Yin F.J., Wang N., Li Y., Cheng B., Wang H.F., Zhai H.B. (2015). A One-Pot Synthesis of Dibenzofurans from 6-Diazo-2-cyclohexenones. Org. Lett..

[10] Zhang C., Shi Y.L., Zhang L.Y., Yuan D.P., Ban M.T., Zheng J.Y., Liu D.H., Guo S.N., Cui D.M. (2018). NaOH-promoted reaction of 1;1-dihaloalkenes and 1*H*-azoles: Synthesis of dihetaryl substituted alkenes. New J. Chem..

[11] Chan D.M.T., Monaco K.L., Wang R.P., Winters M.P. (1998). New *N*- and *O*-arylations with phenylboronic acids and cupric acetate. Tetrahedron Lett..

[12] Evans D.A., Katz J.L., West T.R. (1998). Synthesis of diaryl ethers through the copper-promoted arylation of phenols with arylboronic acids. An expedient synthesis of thyroxine. Tetrahedron Lett..

[13] Doyle M.G., Lundgren R.J. (2021). Oxidative cross-coupling processes inspired by the Chan-Lam reaction. ChemCommun.

[14] Chen T., Xiong H., Yang J.F., Zhu X.L., Qu R.Y., Yang G.F. (2020). Diaryl Ether: A Privileged Scaffold for Drug and Agrochemical Discovery. J. Agric. Food Chem..

[15] Bedos-Belval F., Rouch A., Vanucci-Bacque C., Baltas M. (2012). Diaryl ether derivatives as anticancer agents—A review. MedChemComm.

[16] Pitsinos E.N., Vidali V.P., Couladouros E.A. (2011). Diaryl ether formation in the synthesis of natural products. Eur. J. Org. Chem..

[17] Pan Z.Y., Scheerens H., Li S.J., Schultz B.E., Sprengeler P.A., Burrill L.C., Mendonca R.V., Sweeney M.D., Scott K.C.K., Grothaus P.G. (2007). Discovery of selective irreversible inhibitors for Bruton’s tyrosine kinase. ChemMedChem.

[18] Llovet J.M., Ricci S., Mazzaferro V., Hilgard P., Gane E., Blanc J.F., de Oliveira A.C., Santoro A., Raoul J.L., Forner A. (2008). Sorafenib in advanced hepatocellular carcinoma. N. Engl. J. Med..

[19] Gupta N., Wish J.B. (2017). Hypoxia-inducible factor prolyl hydroxylase inhibitors: A potential new treatment for anemia in patients with CKD. Am. J. Kidney Dis..

[20] Rainsford K.D. (2006). Nimesulide—A multifactorial approach to inflammation and pain: Scientific and clinical consensus. Curr. Med. Res. Opin..

[21] Shu G.W., Yue L., Zhao W.H., Xu C., Yang J., Wang S.B., Yang X.Z. (2015). Isoliensinine; a bioactive alkaloid derived from embryos of Nelumbo nucifera; induces hepatocellular carcinoma cell apoptosis through suppression of NF-κB signaling. J. Agric. Food Chem..

[22] Ikeda R., Che X.F., Yamaguchi T., Ushiyama M., Zheng C.L., Okumura H., Takeda Y., Shibayama Y., Nakamura K., Jeung H.C. (2005). Cepharanthine potently enhances the sensitivity of anticancer agents in K562 cells. Cancer Sci..

[23] Meng Z.P., Li T., Ma X.X., Wang X.Q., Van Ness C., Gan Y.C., Zhou H., Tang J.F., Lou G.Y., Wang Y.F. (2013). Berbamine inhibits the growth of liver cancer cells and cancer-initiating cells by targeting Ca^2+^/calmodulin-dependent protein kinase II. Mol. Cancer Ther..

[24] da Silva A., Maciel D., Freitas V.P., Conserva G.A.A., Alexandre T.R., Purisco S.U., Tempone A.G., Melhem M.S.C., Kato M.J., Guimaraes E.F. (2016). Bioactivity-guided isolation of laevicarpin; an antitrypanosomal and anticryptococcal lactam from Piper laevicarpu (Piperaceae). Fitoterapia.

[25] Hucke O., Coulombe R., Bonneau P., Bertrand-Laperle M., Brochu C., Gillard J., Joly M.A., Landry S., Lepage O., Llinas-Brunet M. (2014). Molecular dynamics simulations and structure-based rational design lead to allosteric HCV NS5B polymerase thumb pocket 2 inhibitor with picomolar cellular replicon potency. J. Med. Chem..

[26] Beaulieu P.L., Coulombe R., Duan J.M., Fazal G., Godbout C., Hucke O., Jakalian A., Joly M.A., Lepage O., Llinas-Brunet M. (2013). Structure-based design of novel HCV NS5B thumb pocket 2 allosteric inhibitors with submicromolar gt1 replicon potency: Discovery of a quinazolinone chemotype. Bioorg. Med. Chem. Lett..

[27] Yang Y.H., Wang Z.L., Yang J.Z., Yang T., Pi W.Y., Ang W., Lin Y.N., Liu Y.Y., Li Z.C., Luo Y.F. (2012). Design, synthesis and evaluation of novel molecules with a diphenyl ether nucleus as potential antitubercular agents. Bioorg. Med. Chem. Lett..

[28] Phainuphong P., Rukachaisirikul V., Phongpaichit S., Preedanon S., Sakayaroj J. (2017). Diphenyl ethers and indanones from the soil-derived fungus Aspergillus unguis PSU-RSPG204. Tetrahedron.

[29] Luemmen P., Kunz K., Greul J., Guth O., Hartmann B., Ilg K., Moradi W.A., Seitz T., Mansfield D., Vors J.P. (2012). Pesticide Phenyloxy Substituted Phenylamidine Derivatives. U.S. Patent.

[30] HRAC (Herbicide Resistance Action Committee). http://www.hracglobal.com.

[31] Dumontet C., Jordan M.A. (2010). Microtubule-binding agents: A dynamic field of cancer therapeutics. Nat. Rev. Drug Discov..

[32] Bates D., Eastman A. (2017). Microtubule destabilising agents: Far more than just antimitotic anticancer drugs. Br. J. Clin. Pharmacol..

[33] Naaz F., Haider M.R., Shafi S., Yar M.S. (2019). Anti-tubulin agents of natural origin: Targeting taxol; vinca; and colchicine binding domains. Eur. J. Med. Chem..

[34] Cao Y.N., Zheng L.L., Wang D., Liang X.X., Gao F., Zhou X.L. (2018). Recent advances in microtubule-stabilizing agents. Eur. J. Med. Chem..

[35] Field J.J., Dıaz J.F., Miller J.H. (2013). The binding sites of microtubule stabilizing agents. Chem. Biol..

[36] Li W., Sun H., Xu S., Zhu Z., Xu J. (2017). Tubulin inhibitors targeting the colchicine binding site: A perspective of privileged structures. Future Med. Chem..

[37] Beale T.M., Allwood D.M., Bender A., Bond P.J., Brenton J.D., Charnock-Jones D.S., Ley S.V., Myers R.M., Shearman J.W., Temple J. (2012). A-ring dihalogenation increases the cellular activity of combretastatin templated tetrazoles. ACS Med. Chem. Lett..

[38] Butenandt A., Wolff A., Karlson P. (1941). Uber Lumi-oestron. Chem. Ber..

[39] Yaremenko F.G., Khvat A.V. (1994). A new one-pot synthesis of 17-oxo-13α-steroids of the androstane series from their 13β-analogues. Mendeleev Commun..

[40] Ayan D., Roy J., Maltais R., Poirier D. (2011). Impact of estradiol structural modifications (18-methyl and/or 17-hydroxy inversion of configuration) on the in vitro and in vivo estrogenic activity. J. Steroid Biochem. Mol. Biol..

[41] Schonecker B., Lange C., Kotteritzsch M., Gunther W., Weston J., Anders E., Gorls H. (2000). Conformational design for 13α-steroids. J. Org. Chem..

[42] Szabo J., Pataki Z., Wolfling J., Schneider G., Bózsity N., Minorics R., Zupkó I., Mernyák E. (2016). Synthesis and biological evaluation of 13a-estrone derivatives as potential antiproliferative agents. Steroids.

[43] Bacsa I., Herman B.E., Jojart R., Herman K.S., Wölfling J., Schneider G., Varga M., Tömböly C., Rižner T.L., Szécsi M. (2018). Synthesis and structure–activity relationships of 2- and/or 4-halogenated 13β- and 13α-estrone derivatives as enzyme inhibitors of estrogen biosynthesis. J. Enzym. Inhib. Med. Chem..

[44] Jojart R., Pecsy S., Keglevich G., Szécsi M., Rigó R., Özvegy-Laczka C., Kecskeméti G., Mernyák E. (2018). Pd-catalyzed microwave-assisted synthesis of phosphonated 13α-estrones as potential OATP2B1; 17β-HSD1 and/or STS inhibitors. Beilstein J. Org. Chem..

[45] Sinreih M., Jójárt R., Kele Z., Büdefeld T., Paragi G., Mernyák E., Rižner T.L. (2021). Synthesis and evaluation of AKR1C inhibitory properties of A-ring halogenated oestrone derivatives. J. Enzym. Inhib. Med. Chem..

[46] Jójárt R., Laczkó-Rigó R., Klement M., Kőhl G., Kecskeméti G., Özvegy-Laczka C., Mernyák E. (2021). Design, synthesis and biological evaluation of novel estrone phosphonates as high affinity organic anion-transporting polypeptide 2B1 (OATP2B1) inhibitors. Bioorg. Chem..

[47] Traj P., Abdolkhaliq A.H., Németh A., Dajcs S.T., Tömösi F., Lanisnik-Rizner T., Zupkó I., Mernyák E. (2021). Transition metal-catalysed A-ring C-H activations and C(sp^2^)-C(sp^2^) couplings in the 13α-oestrone series and in vitro evaluation of antiproliferative properties. J. Enzym. Inhib. Med. Chem..

[48] Jójárt R., Tahaei S.A.S., Trungel-Nagy P., Kele Z., Minorics R., Paragi G., Zupkó I., Mernyák E. (2021). Synthesis and evaluation of anticancer activities of 2- or 4-substituted 3-(N-benzyltriazolylmethyl)-13α-oestrone derivatives. J. Enzym. Inhib. Med. Chem..

[49] Mosmann T. (1983). Rapid colorimetric assay for cellular growth and survival: Application to proliferation and cytotoxicity assays. J. Immunol. Methods.

[50] Lee Y.K., Lim J., Yoon S.Y., Joo J.C., Park S.J., Park Y.J. (2019). Promotion of cell death in cisplatin-resistant ovarian cancer cells through KDM1BDCLRE1B modulation. Int. J. Mol. Sci..

[51] Schelz Z., Ocsovszki I., Bozsity N., Hohmann J., Zupkó I. (2016). Antiproliferative effects of various furanoacridones isolated from Ruta graveolens on human breast cancer cell lines. Anticancer Res..

[52] Chavez K.J., Garimella S.V., Lipkowitz S. (2010). Triple negative breast cancer cell lines: One tool in the search for better treatment of triple negative breast cancer. Breast Dis..

[53] Anders C.K., Zagar T.M., Carey L.A. (2013). The management of earlystage and metastatic triple-negative breast cancer: A review. Hematol. Oncol. Clin. N. Am..

[54] Suba Z. (2014). Triple-negative breast cancer risk in women is defined by the defect of estrogen signaling: Preventive and therapeutic implications. Onco Targets Ther..

[55] Goodman A. (2015). HPV testing as a screen for cervical cancer. BMJ.

[56] Ghittoni R., Accardi R., Chiocca S., Tommasino M. (2015). Role of human papillomaviruses in carcinogenesis. Ecancermedicalscience.

[57] Kellogg E.H., Hejab N.M., Howes S., Northcote P., Miller J.H., Diaz J.F., Downing K.H., Nogales E. (2017). Insights into the Distinct Mechanisms of Action of Taxane and Non-Taxane Microtubule Stabilizers from Cryo-EM Structures. J. Mol. Biol..

[58] Schrödinger Release 2021-3: Protein Preparation Wizard; Epik, Schrödinger, LLC, New York, NY, USA, 2021; Impact, Schrödinger, LLC, New York, NY; Prime, Schrödinger, LLC, New York, NY, USA, 2021.

[59] Maestro Schrödinger Release 2021-3: Maestro, Schrödinger, LLC, New York, NY, USA, 2021.

[60] Schrödinger Release 2021-3: Glide, Schrödinger, LLC, New York, NY, USA, 2021.

[61] Schrödinger Release 2021-3: Desmond Molecular Dynamics System, D. E. Shaw Research, New York, NY, USA, 2021. Maestro-Desmond Interoperability Tools, Schrödinger, New York, NY, USA, 2021.

[62] Lu C., Wu C., Ghoreishi D., Chen W., Wang L., Damm W., Ross G., Dahlgren M., Russell E., Von Bargen C. (2021). OPLS4: Improving Force Field Accuracy on Challenging Regimes of Chemical Space. J. Chem. Theory Comput..

