# Supplementary material for: Synthesis and Antiproliferative Activity of Steroidal Diaryl Ethers
Source: Molecules. 2023 Jan 25;28(3):1196. doi: 10.3390/molecules28031196 (PMC9919549; doi:10.3390/molecules28031196)
Supplement: Supplementary file 1 [file molecules-28-01196-s001.zip › molecules-2148376-supplementary.pdf]

Synthesis and antiproliferative activity of steroidal diaryl ethers

Édua Kovács,[1] Hazhmat Ali,[2] Renáta Minorics,[2] Péter Traj,[1] Vivien Resch,[3] Gábor Paragi,[3,4,5] Bella Bruszel,[3] István Zupkó,[2] Erzsébet Mernyák[1]*

[1]Department of Organic Chemistry, University of Szeged, H-6720 Szeged, Hungary

[2]Institute of Pharmacodynamics and Biopharmacy, University of Szeged, H-6720 Szeged, Hungary

[3]Department of Medicinal Chemistry, University of Szeged, H-6720 Szeged, Hungary

[4]Institute of Physics, University of Pécs, H-7625, Pécs, Hungary

[5]Department of Theoretical Physics, University of Szeged, H-6720, Szeged, Hungary

*Corresponding author. Tel.: +36-62-544277; fax: +36-62-544200 (E. Mernyák)

E-mail address: bobe@chem.u-szeged.hu (E. Mernyák)

Table S1. Antiproliferative activities of compounds **14a–l**

Compound	Conc.	MCF-7	MDA-MB -231	HeLa	SiHa	A2780	NIH/3T3
**14a**	10 µM						
	30 µM						
**14b**	10 µM						
	30 µM						
**14c**	10 µM						
	30 µM						
**14d**	10 µM						
	30 µM						
**14e**	10 µM						
	30 µM						
**14f**	10 µM						
	30 µM						
**14g**	10 µM						
	30 µM						
**14h**	10 µM						
	30 µM						
**14i**	10 µM						
	30 µM						
**14j**	10 µM						
	30 µM						
**14k**	10 µM						
	30 µM						
**14l**	10 µM						
	30 µM						
**cisplatin**	10 µM						
	30 µM						

Cancer cell growth inhibition: 20% or less

Cancer cell growth inhibition: 100%

Figure S1. Presence of secondary interactions between the protein amino acids and **14i** ligand along the 1st trajectory.

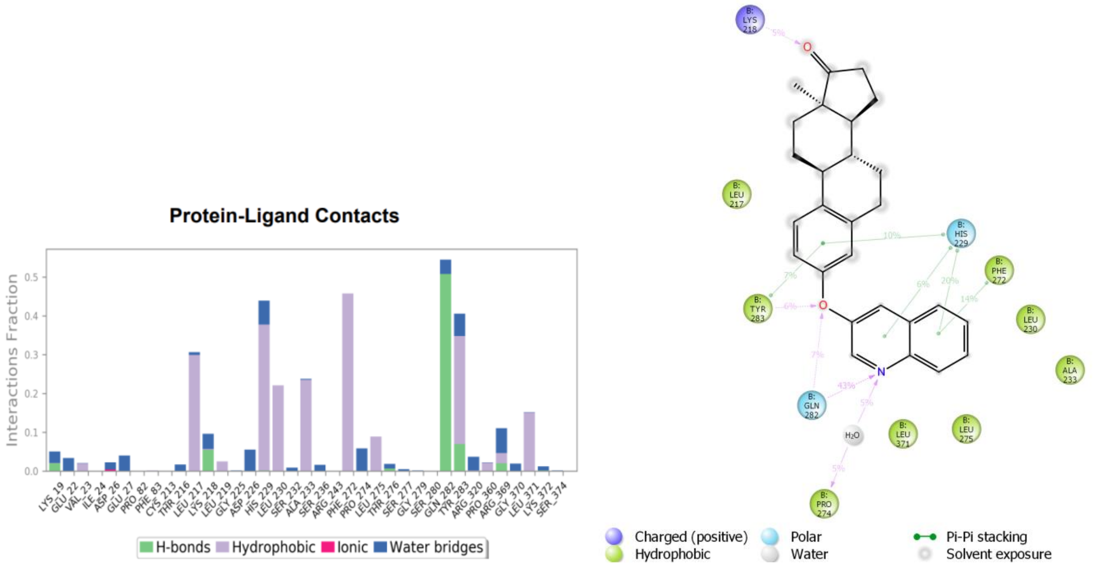

Figure S2. Presence of secondary interactions between the protein amino acids and **14i** ligand along the 2nd trajectory.

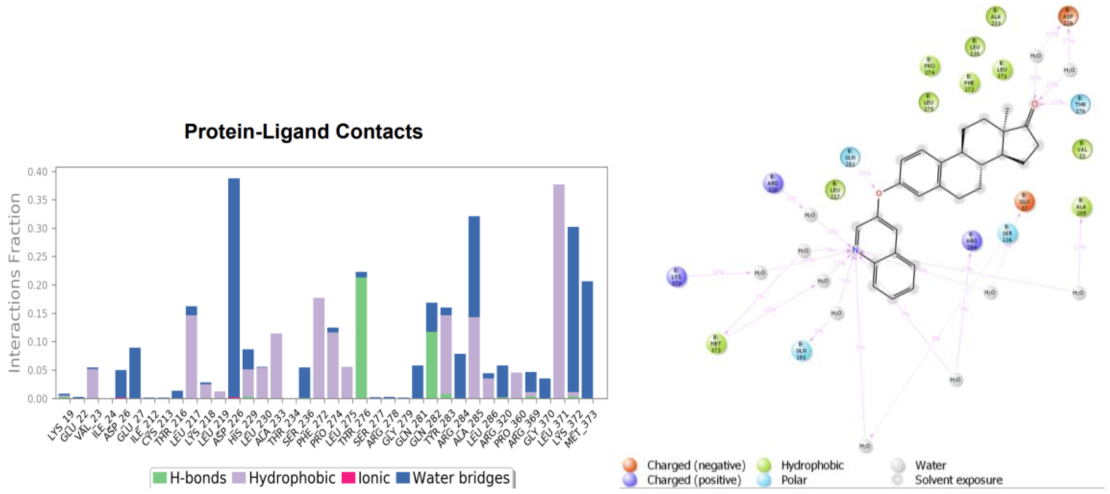

Figure S3. Presence of secondary interactions between the protein amino acids and **14i** ligand along the 3rd trajectory.

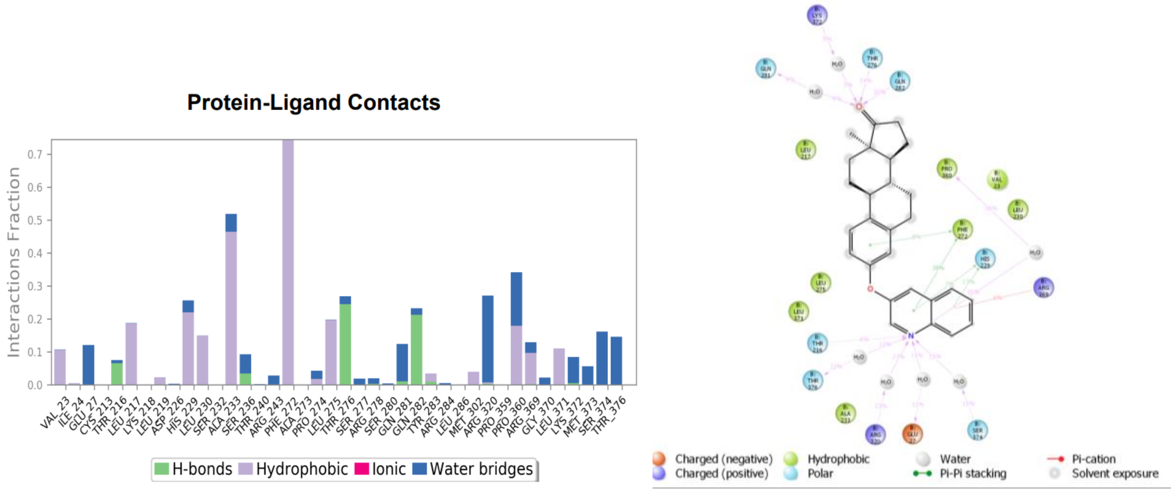

Figure S4. Presence of secondary interactions between the protein amino acids and **14i** ligand along the 4th trajectory.

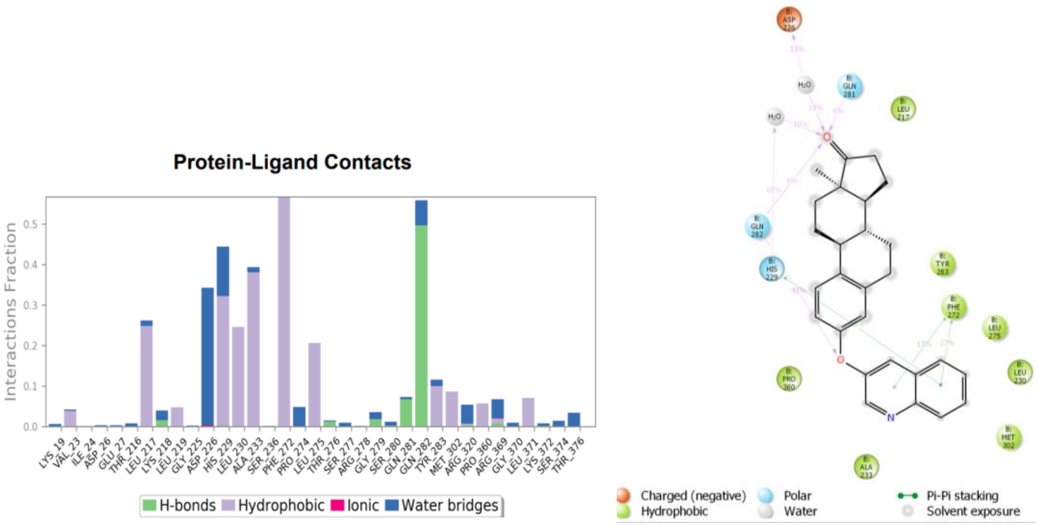

Figure S5. Presence of secondary interactions between the protein amino acids and **14i** ligand along the 5th trajectory.

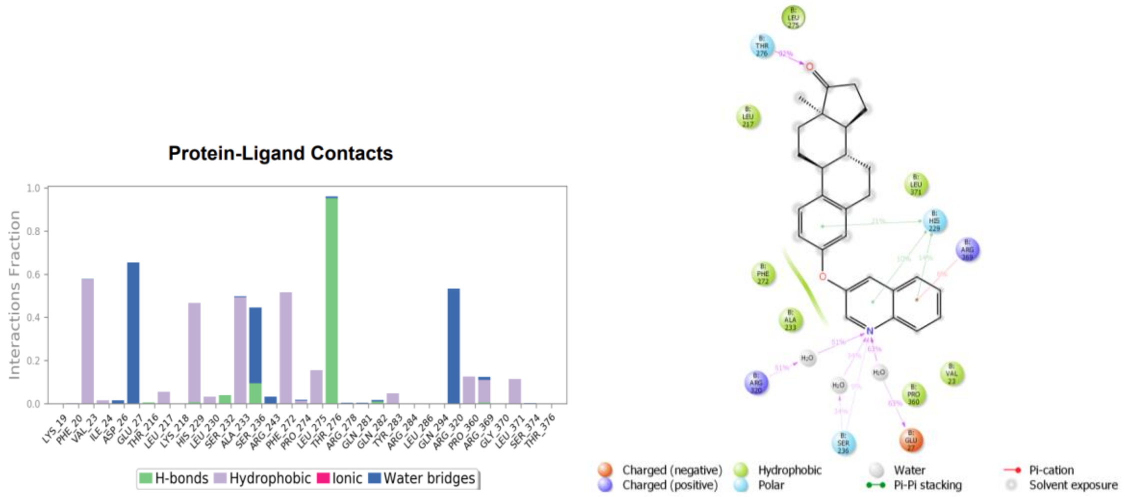

^{1}H and ^{13}C NMR spectra of the newly synthesized compounds.

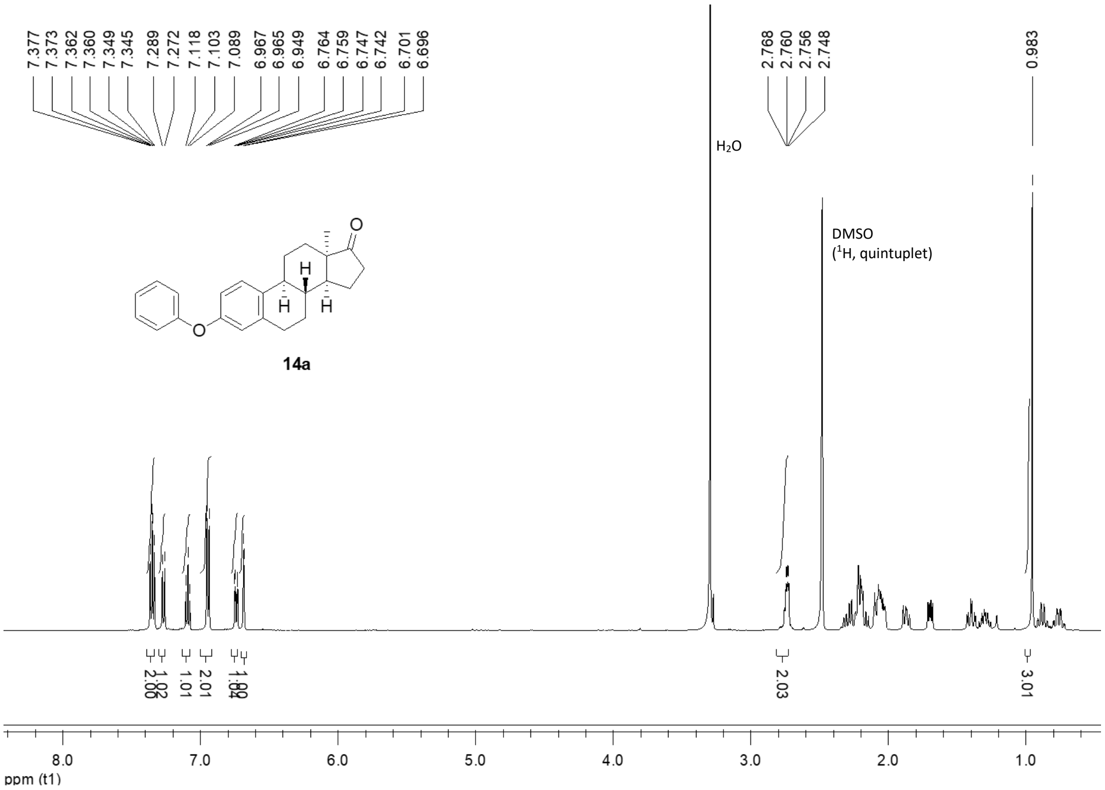

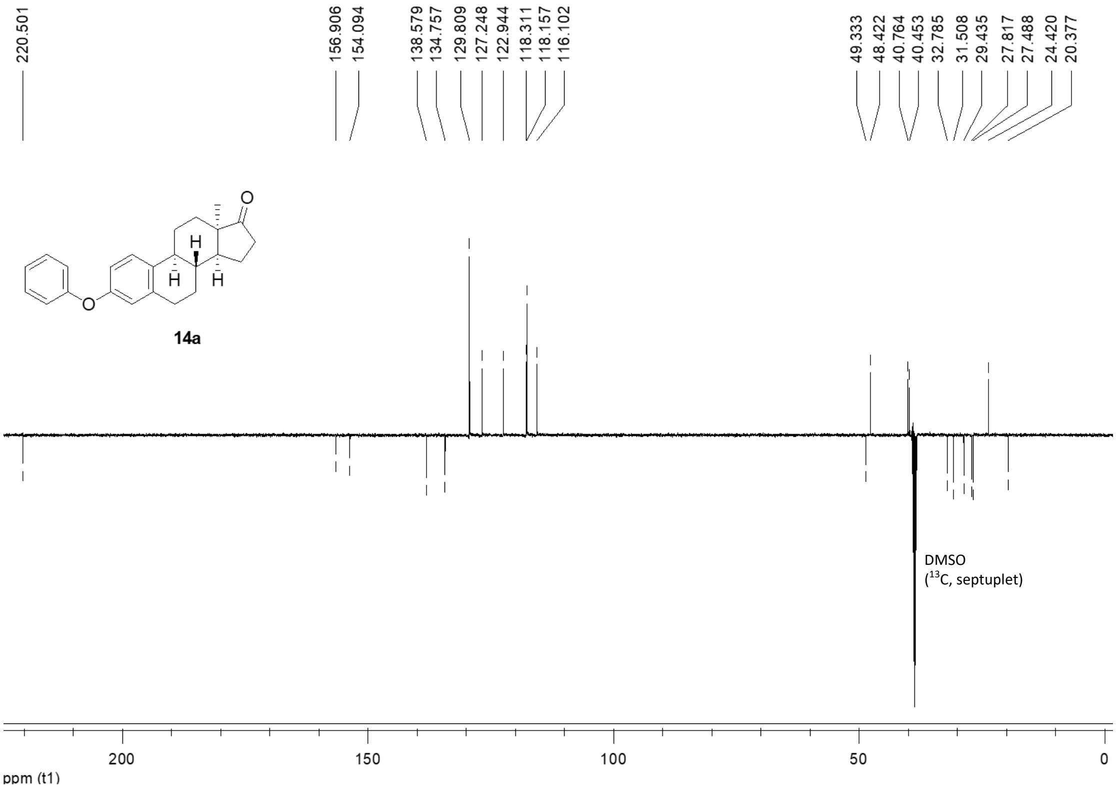

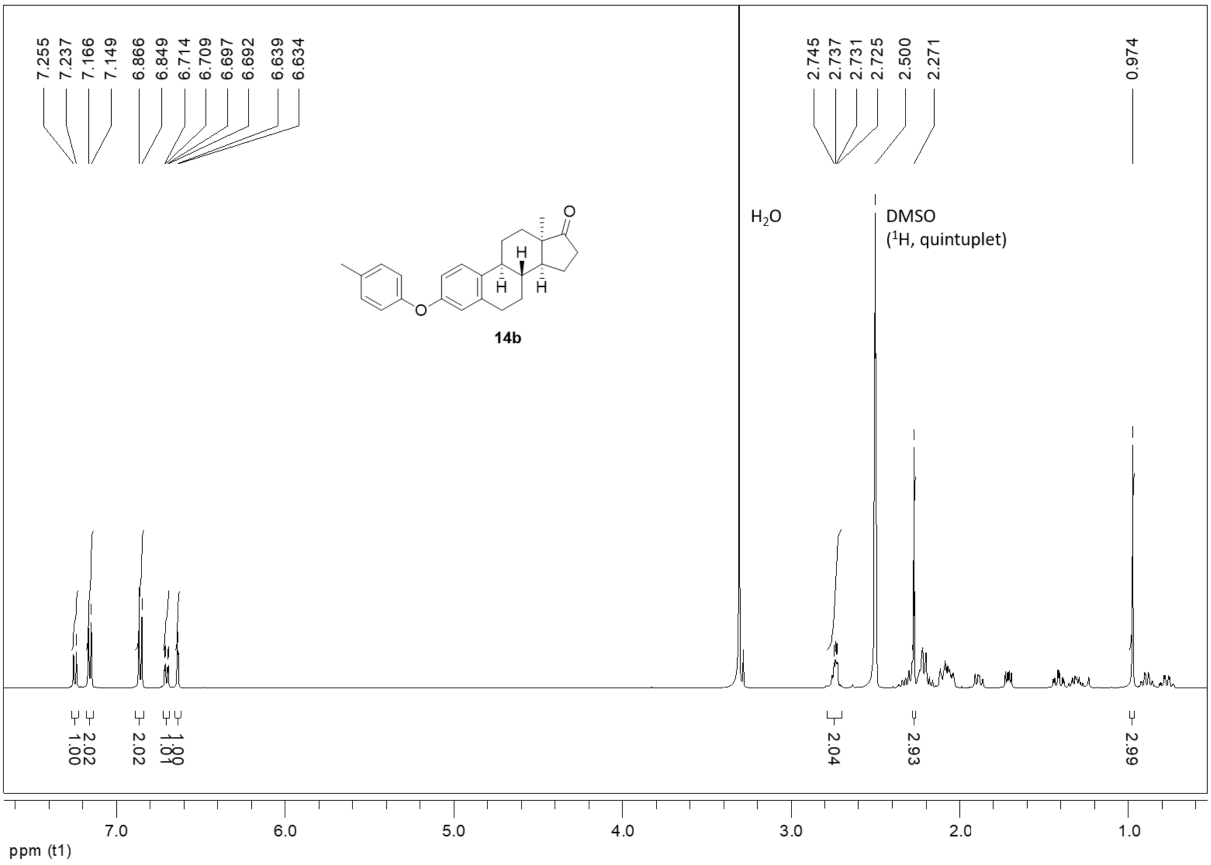

7.255
7.237
7.166
7.149
6.866
6.849
6.714
6.709
6.697
6.692
6.639
6.634
2.745
2.737
2.731
2.725
2.500
2.271
0.974
H2O
DMSO
(1H, quintuplet)
14b
1.00
2.02
2.02
1.00
1.01
2.04
2.93
2.99
7.0
6.0
5.0
4.0
3.0
2.0
1.0
ppm (t1)

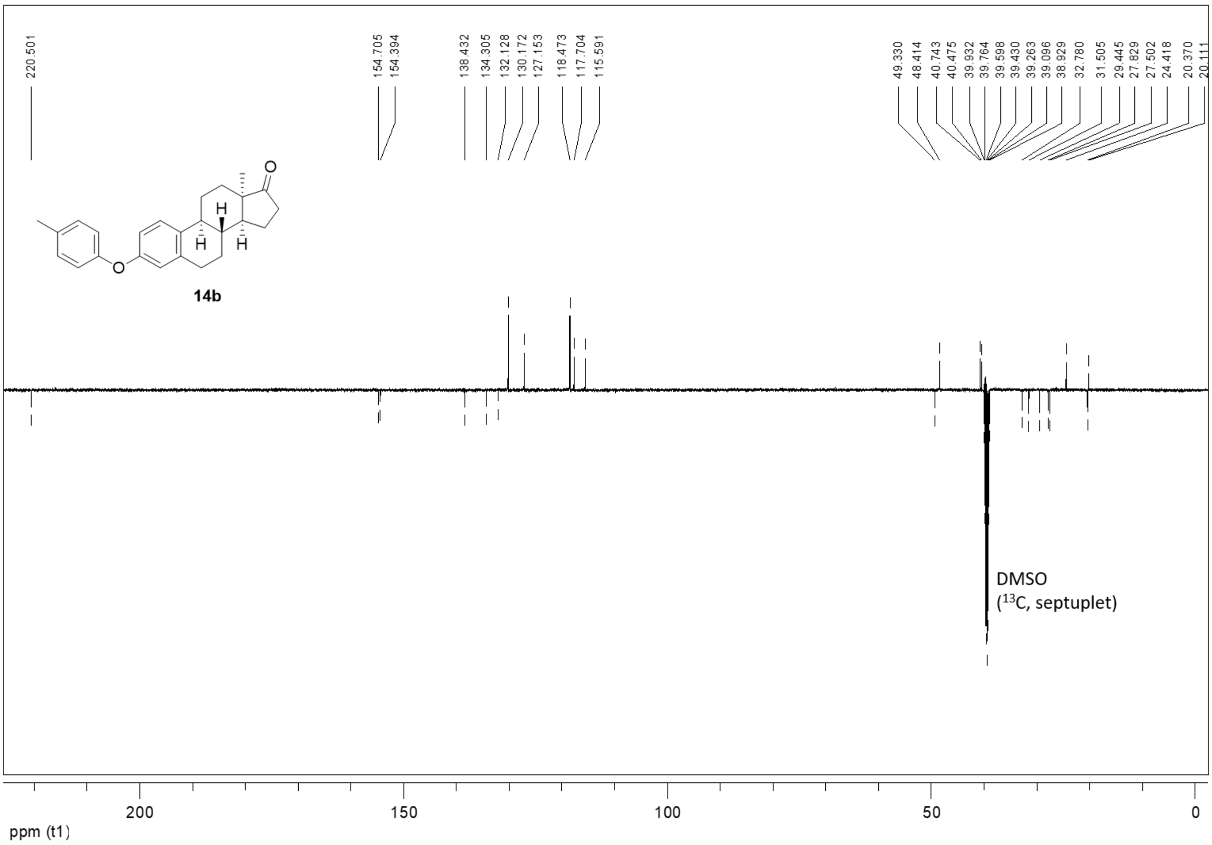

220.501
154.705
154.394
138.432
134.305
132.128
130.172
127.153
118.473
117.704
115.591
49.330
48.414
40.743
40.475
39.932
39.764
39.598
39.430
39.263
39.096
38.929
32.780
31.505
29.445
27.829
27.502
24.418
20.370
20.111
14b
DMSO
(13C, septuplet)
200
150
100
50
0
ppm (t1)

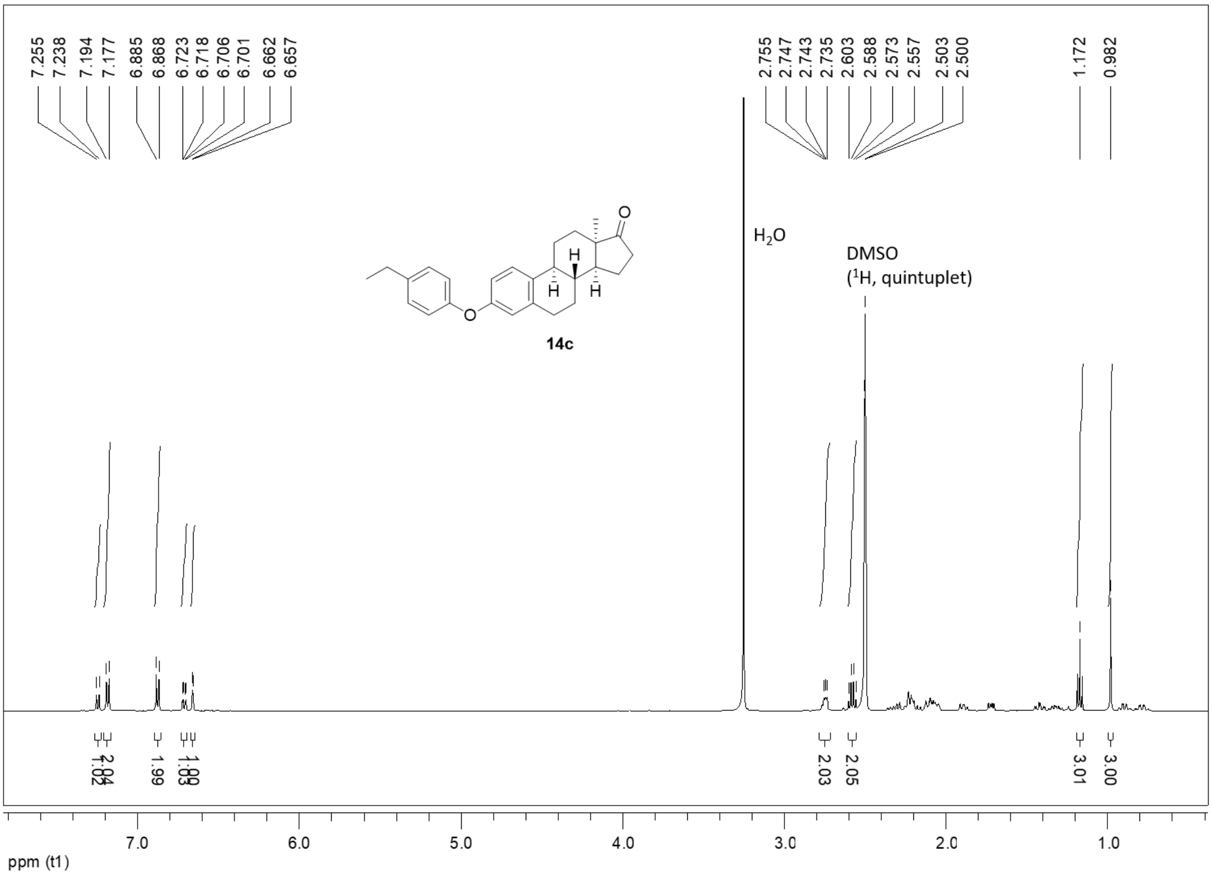
7.255
7.238
7.194
7.177
6.885
6.868
6.723
6.718
6.706
6.701
6.662
6.657
2.755
2.747
2.743
2.735
2.603
2.588
2.573
2.557
2.503
2.500
1.172
0.982
H_2O
DMSO
(1H, quintuplet)
14c
1.99
2.03
2.05
3.01
3.00
7.0
6.0
5.0
4.0
3.0
2.0
1.0
ppm (t1)

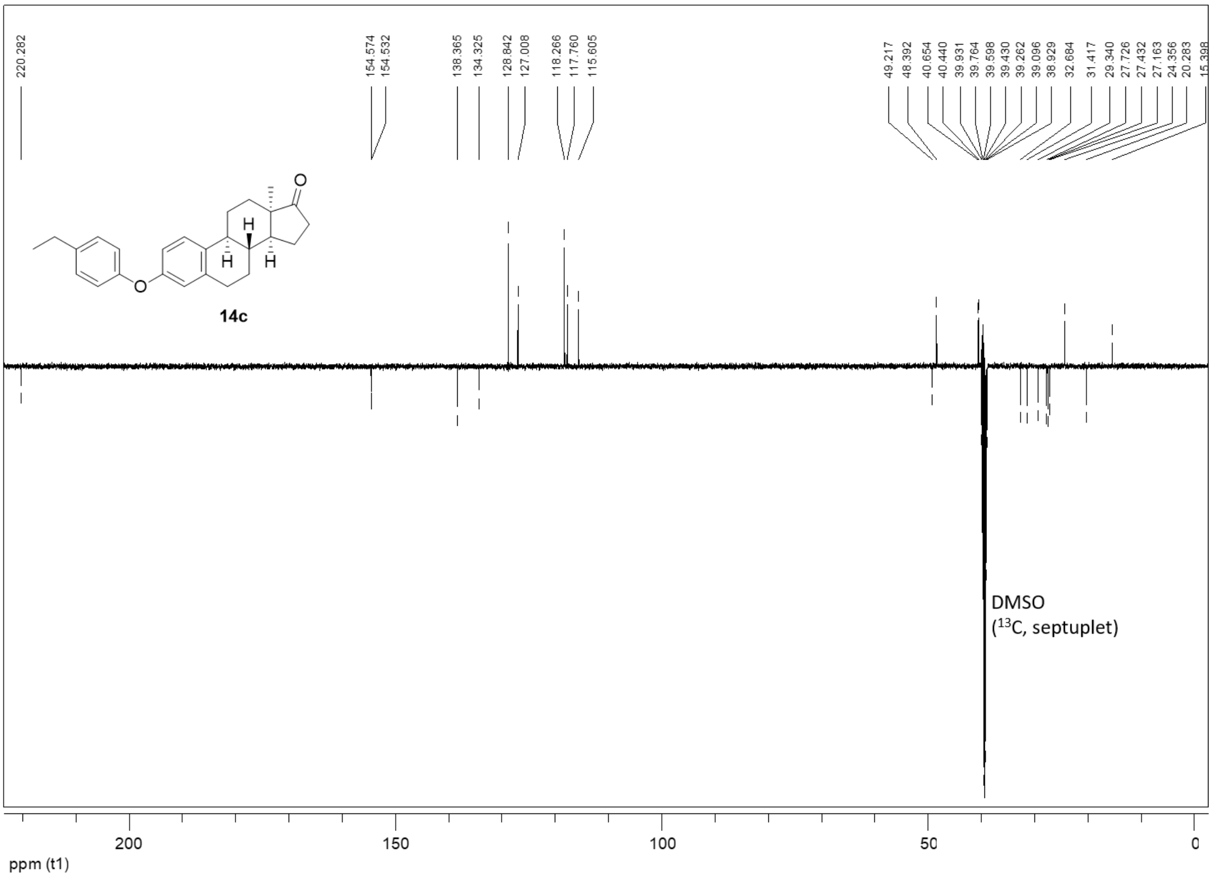
220.282
154.574
154.532
138.365
134.325
128.842
127.008
118.266
117.760
115.605
49.217
48.392
40.654
40.440
39.931
39.764
39.598
39.430
39.262
39.096
38.929
32.684
31.417
29.340
27.726
27.432
27.163
24.356
20.283
15.398
14c
DMSO
(13C, septuplet)
200
150
100
50
0
ppm (t1)

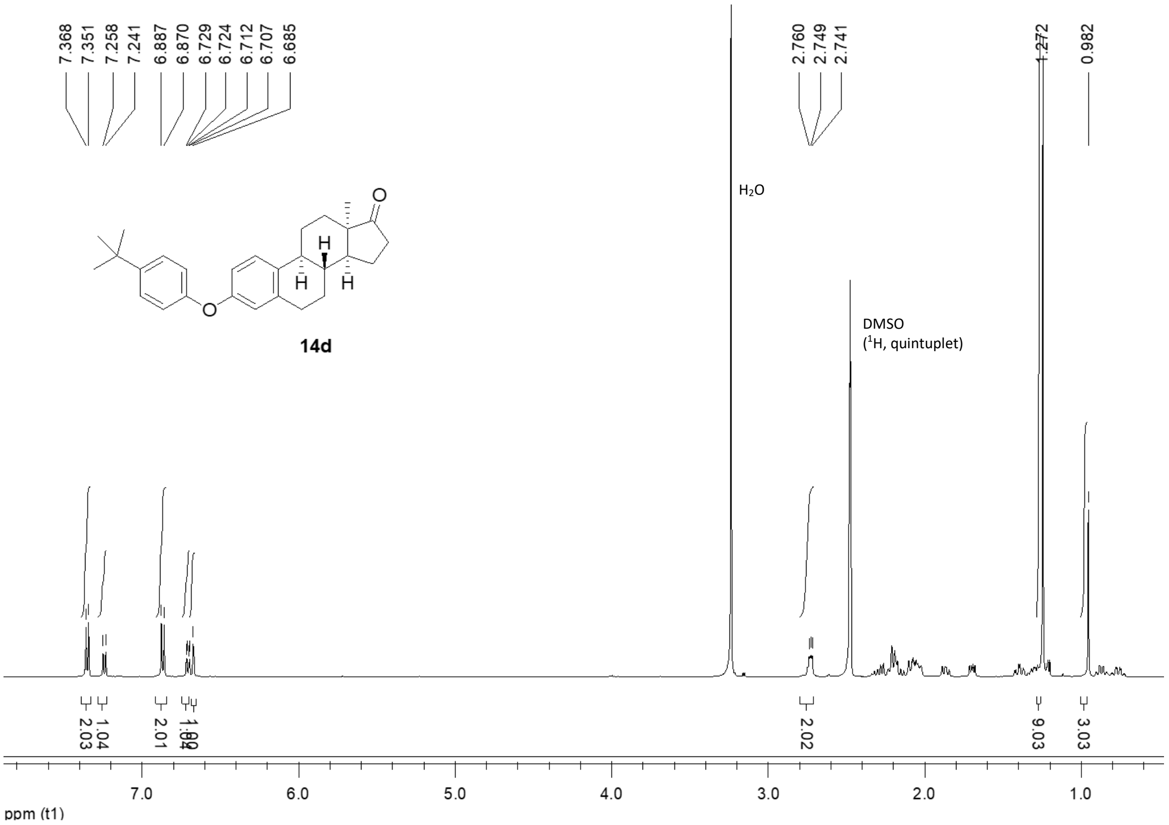
7.368
7.351
7.258
7.241
6.887
6.870
6.729
6.724
6.712
6.707
6.685
2.760
2.749
2.741
0.982
H$_2$O
14d
DMSO
(^{1}H, quintuplet)
2.03
1.04
2.01
2.02
9.03
3.03
7.0
6.0
5.0
4.0
3.0
2.0
1.0
ppm (t1)

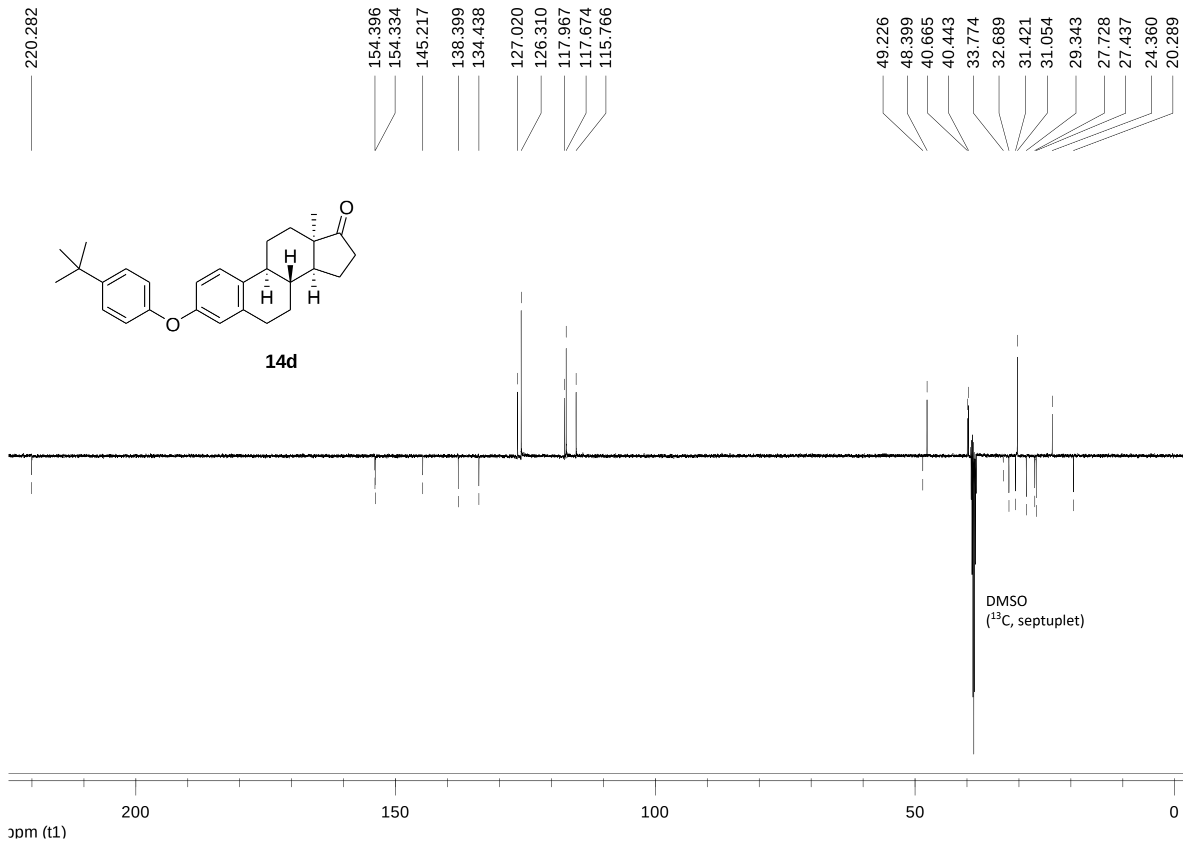
220.282
154.396
154.334
145.217
138.399
134.438
127.020
126.310
117.967
117.674
115.766
49.226
48.399
40.665
40.443
33.774
32.689
31.421
31.054
29.343
27.728
27.437
24.360
20.289
14d
DMSO
(^{13}C, septuplet)
200
150
100
50
0

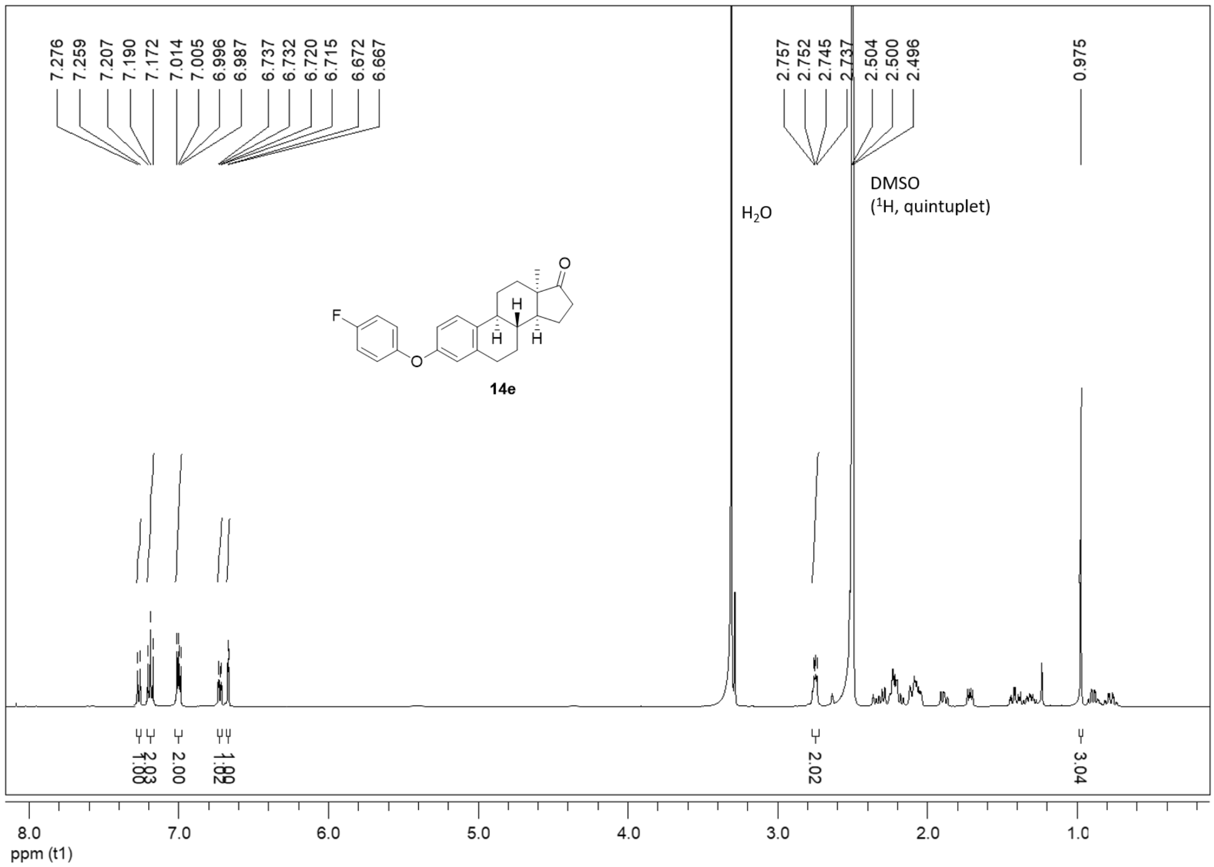
7.276
7.259
7.207
7.190
7.172
7.014
7.005
6.996
6.987
6.737
6.732
6.720
6.715
6.672
6.667
2.757
2.752
2.745
2.737
2.504
2.500
2.496
0.975
H2O
DMSO
(1H, quintuplet)
F
O
H
14e
2.00
2.02
3.04
8.0
7.0
6.0
5.0
4.0
3.0
2.0
1.0
ppm (t1)

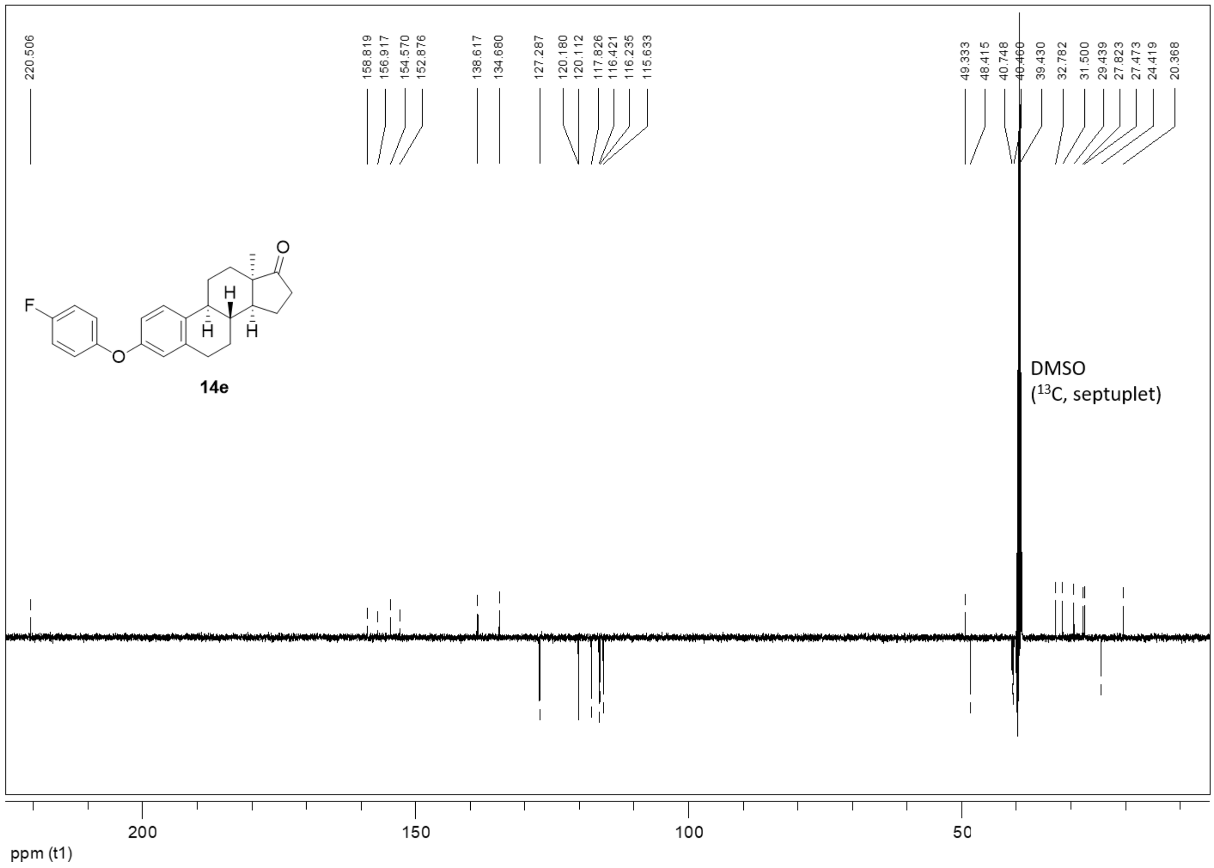
220.506
158.819
156.917
154.570
152.876
138.617
134.680
127.287
120.180
120.112
117.826
116.421
116.235
115.633
49.333
48.415
40.748
39.430
32.782
31.500
29.439
27.823
27.473
24.419
20.368
F
O
H
14e
DMSO
(13C, septuplet)
200
150
100
50
ppm (t1)

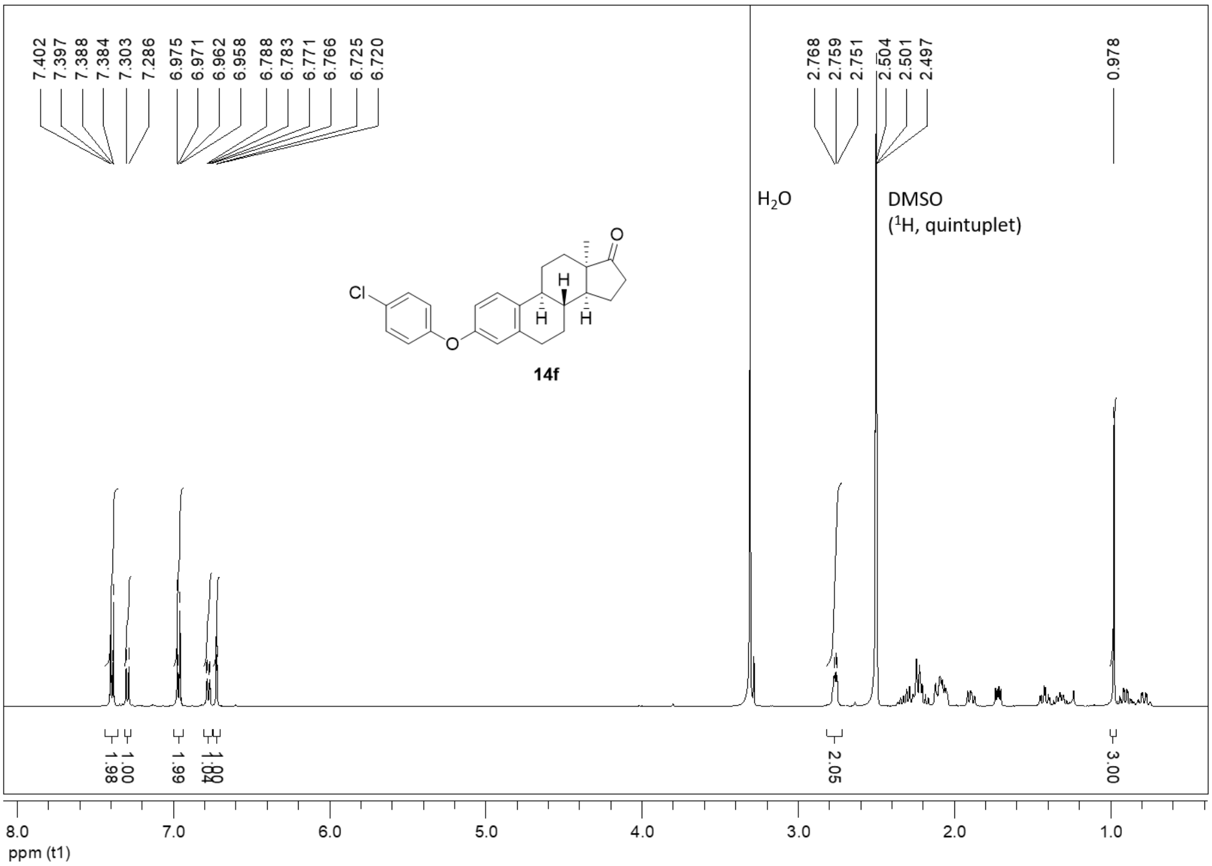

7.402
7.397
7.388
7.384
7.303
7.286
6.975
6.971
6.962
6.958
6.788
6.783
6.771
6.766
6.725
6.720
2.768
2.759
2.751
2.504
2.501
2.497
0.978
H2O
DMSO
(1H, quintuplet)
Cl
O
H
14f
1.98
1.00
1.99
1.04
1.00
2.05
3.00
8.0
7.0
6.0
5.0
4.0
3.0
2.0
1.0
ppm (t1)

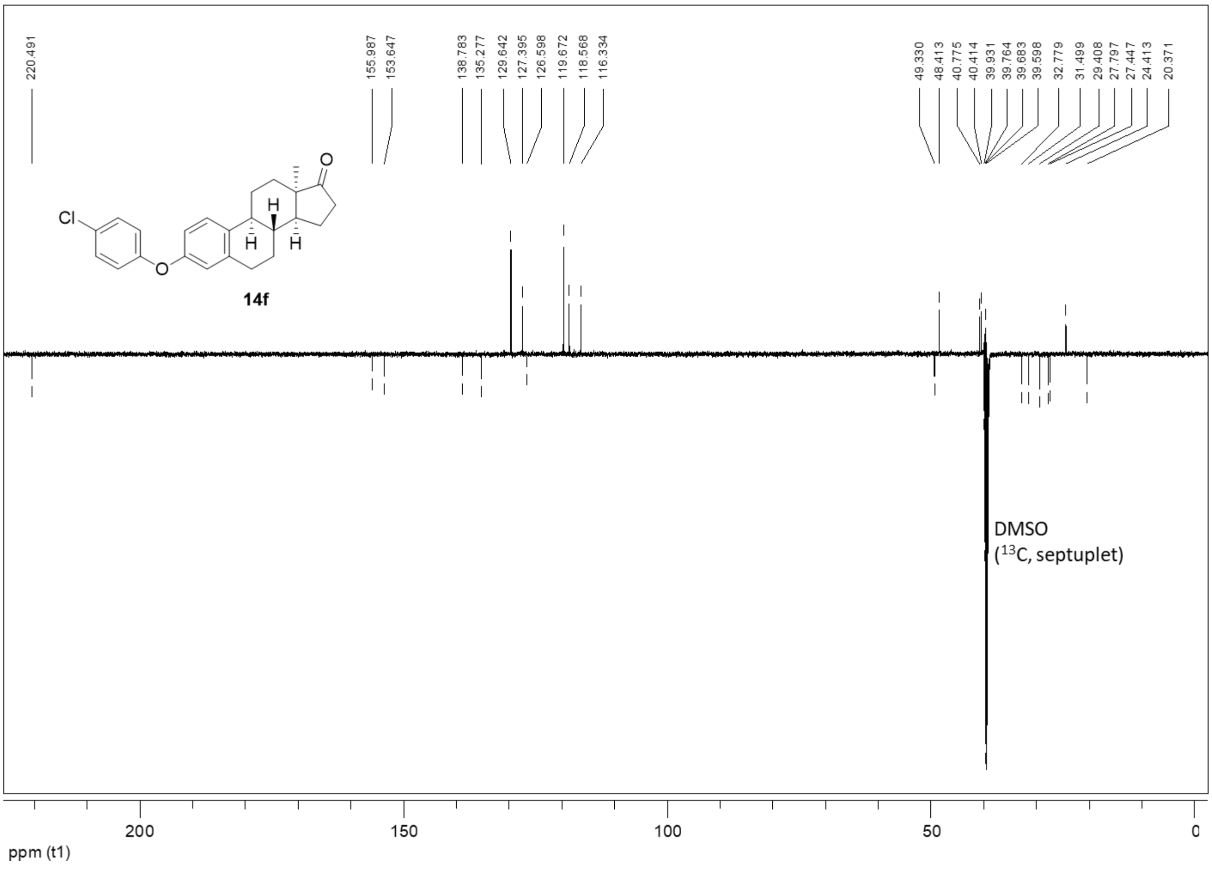

220.491
155.987
153.647
138.783
135.277
129.642
127.395
126.598
119.672
118.568
116.334
49.330
48.413
40.775
40.414
39.931
39.764
39.683
39.598
32.779
31.499
29.408
27.797
27.447
24.413
20.371
Cl
O
H
14f
DMSO
(13C, septuplet)
200
150
100
50
0
ppm (t1)

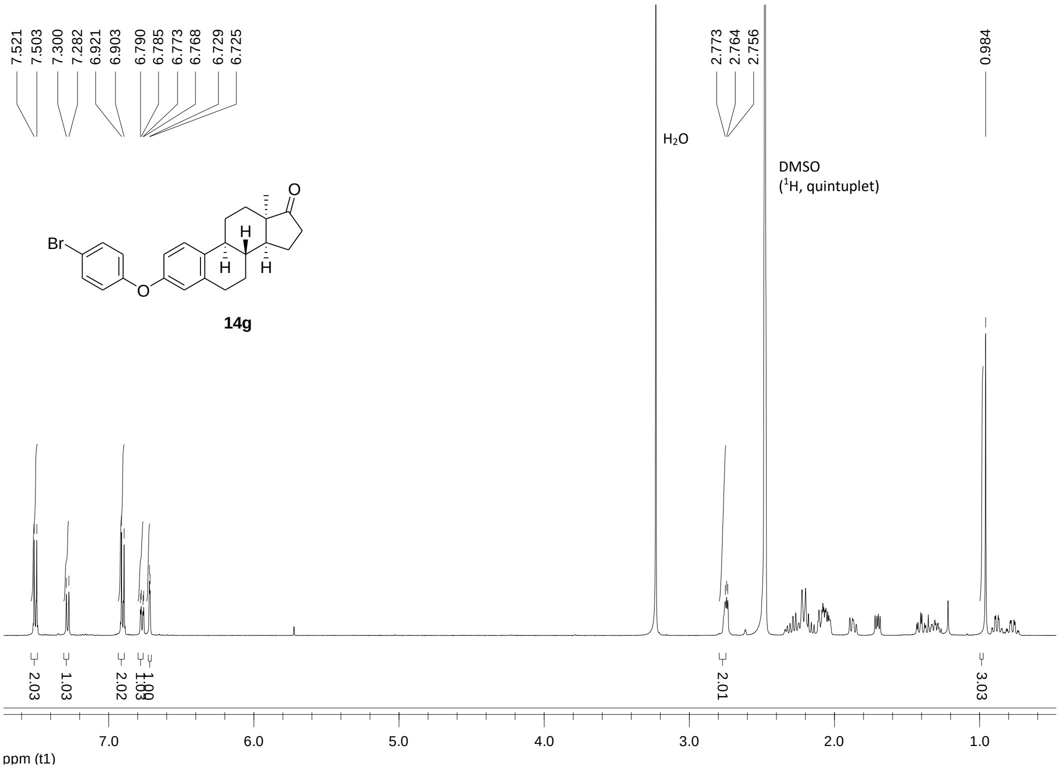
7.521
7.503
7.300
7.282
6.921
6.903
6.790
6.785
6.773
6.768
6.729
6.725
2.773
2.764
2.756
0.984
H$_2$O
DMSO
(^{1}H, quintuplet)
Br
O
H
14g
2.03
1.03
2.02
2.01
3.03
7.0
6.0
5.0
4.0
3.0
2.0
1.0
ppm (t1)

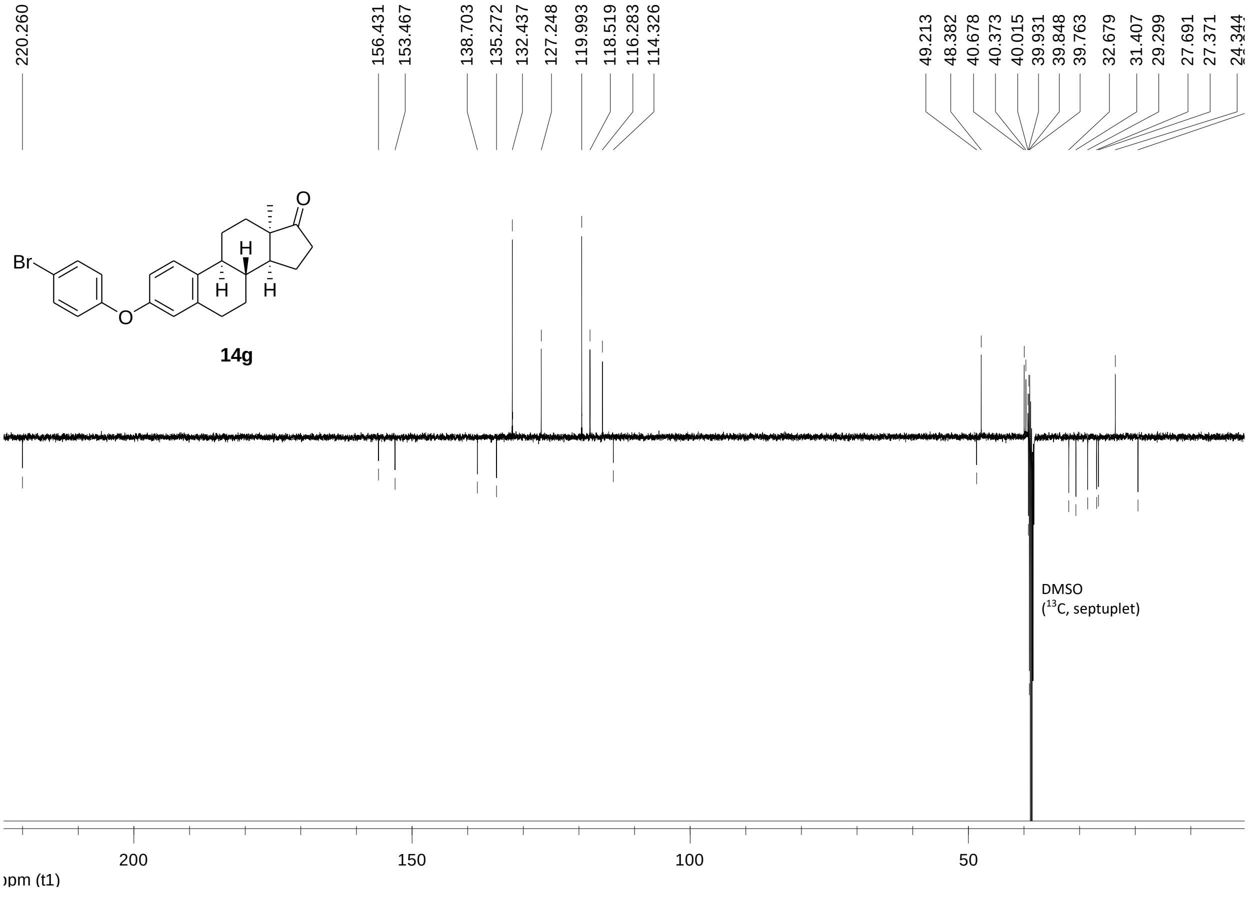
220.260
156.431
153.467
138.703
135.272
132.437
127.248
119.993
118.519
116.283
114.326
49.213
48.382
40.678
40.373
40.015
39.931
39.848
39.763
32.679
31.407
29.299
27.691
27.371
24.344
Br
O
H
14g
DMSO
(^{13}C, septuplet)
200
150
100
50
ppm (t1)

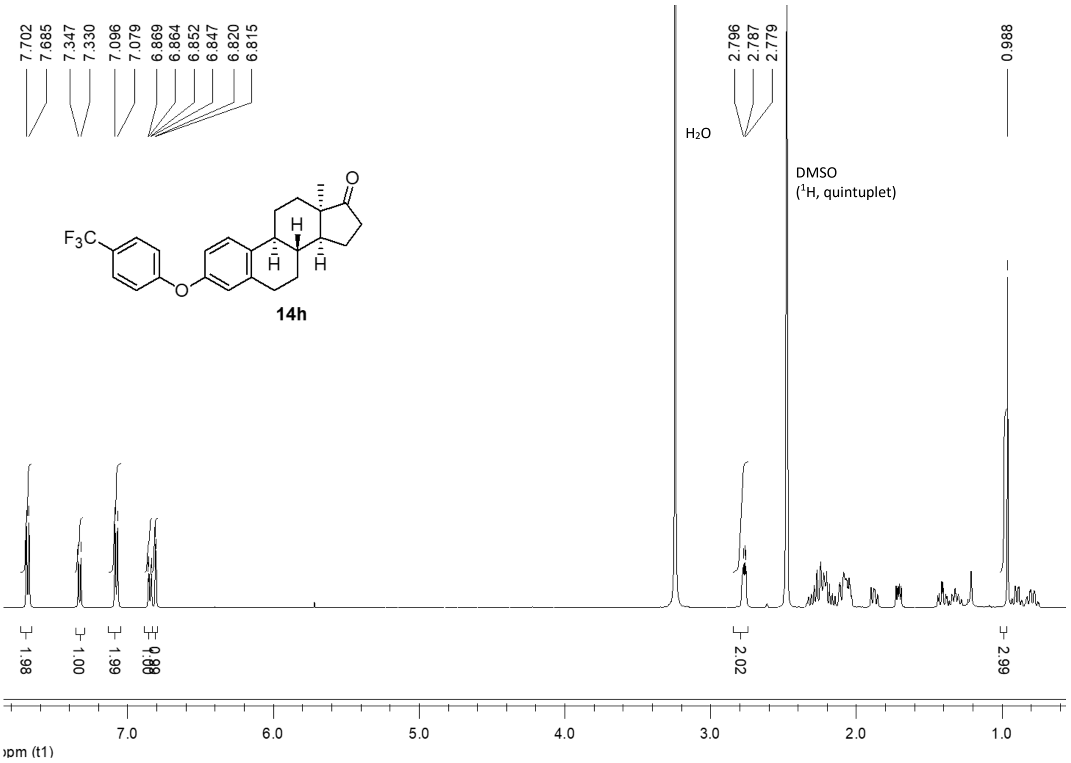
7.702
7.685
7.347
7.330
7.096
7.079
6.869
6.864
6.852
6.847
6.820
6.815
2.796
2.787
2.779
0.988
H2O
DMSO
(1H, quintuplet)
F3C
O
H
H
H
O
14h
1.98
1.00
1.99
2.02
2.99
7.0
6.0
5.0
4.0
3.0
2.0
1.0
ppm (t1)

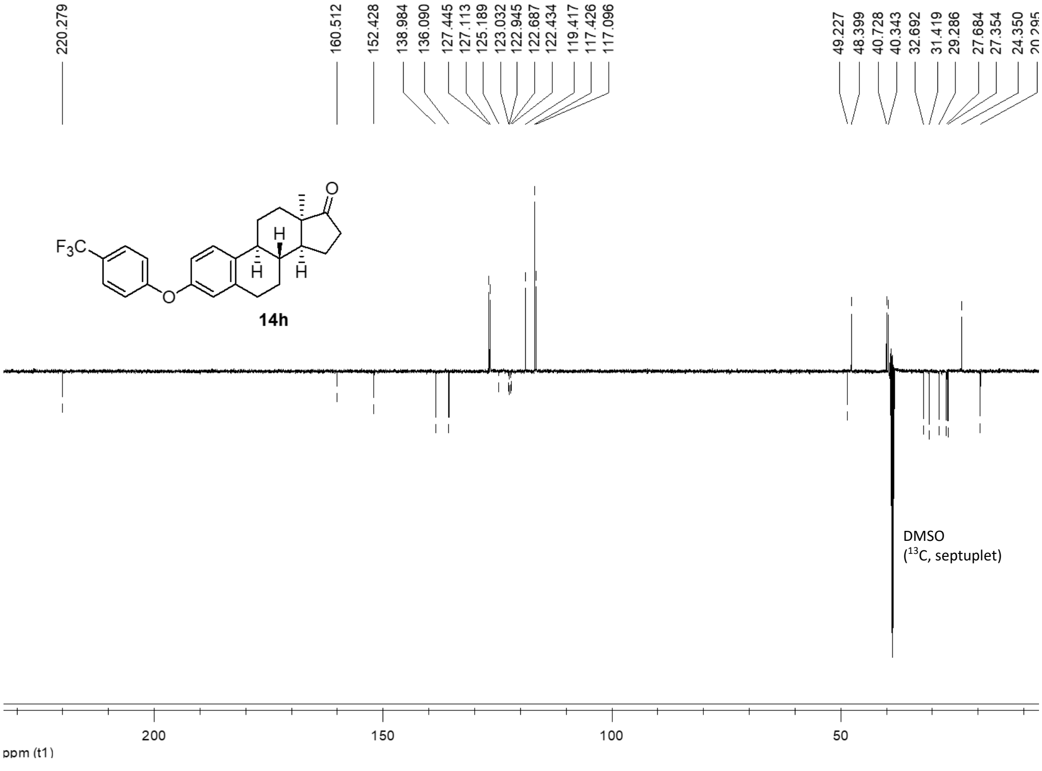
220.279
160.512
152.428
138.984
136.090
127.445
127.113
125.189
123.032
122.945
122.687
122.434
119.417
117.426
117.096
49.227
48.399
40.728
40.343
32.692
31.419
29.286
27.684
27.354
24.350
F3C
O
H
H
H
O
14h
DMSO
(13C, septuplet)
200
150
100
50
ppm (t1)

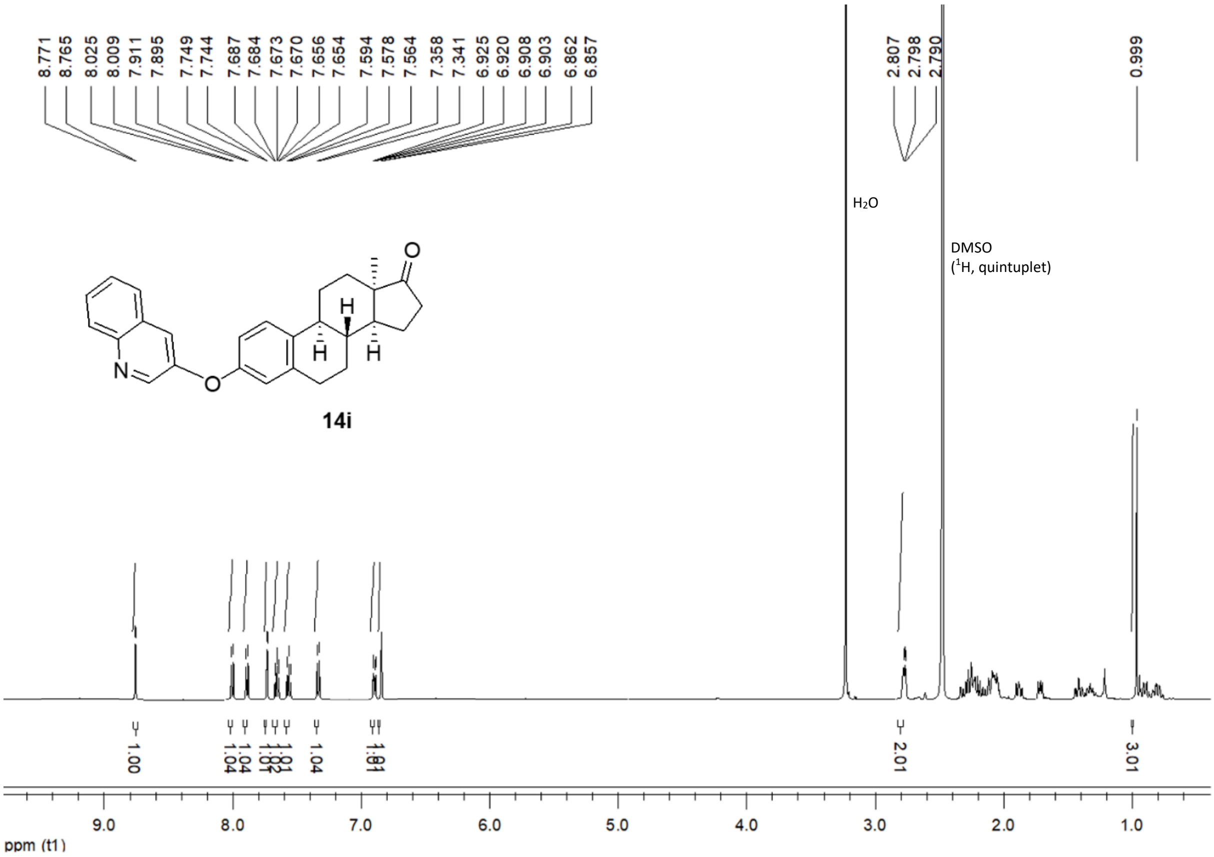
8.771 8.765 8.025 8.009 7.911 7.895 7.749 7.744 7.687 7.684 7.673 7.670 7.656 7.654 7.594 7.578 7.564 7.358 7.341 6.925 6.920 6.908 6.903 6.862 6.857
2.807 2.798 2.790
0.999
H2O
DMSO
(1H, quintuplet)
14i
1.00 1.04 1.04 1.04 2.01 3.01
9.0 8.0 7.0 6.0 5.0 4.0 3.0 2.0 1.0
ppm (t1)

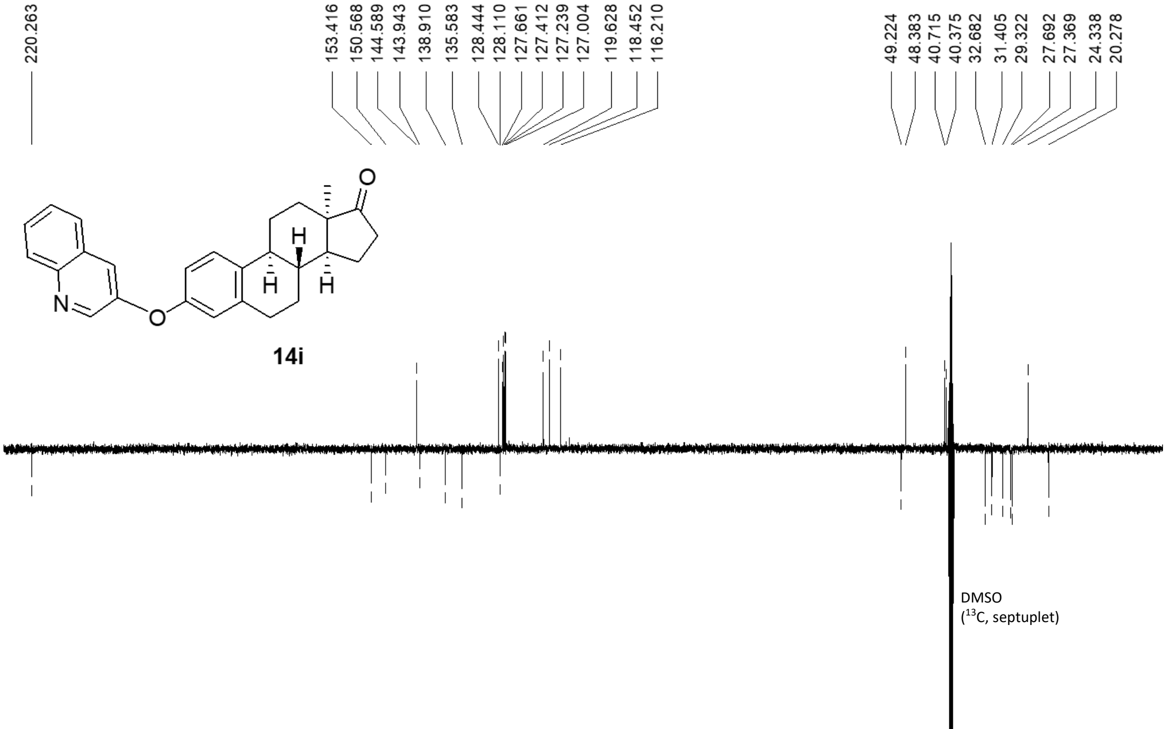
220.263
153.416 150.568 144.589 143.943 138.910 135.583 128.444 128.110 127.661 127.412 127.239 127.004 119.628 118.452 116.210
49.224 48.383 40.715 40.375 32.682 31.405 29.322 27.692 27.369 24.338 20.278
14i
DMSO
(13C, septuplet)
200 150 100 50 0
ppm (t1)

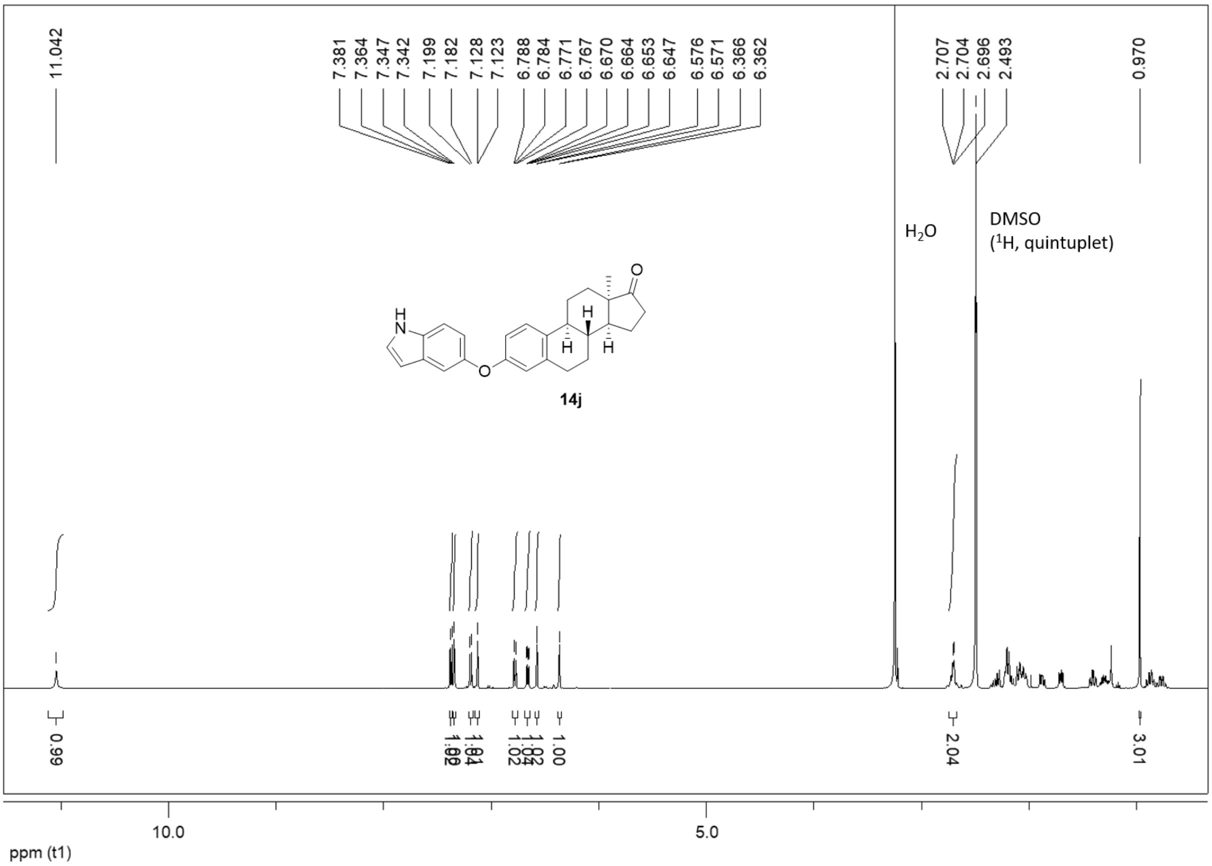

11.042
7.381
7.364
7.347
7.342
7.199
7.182
7.128
7.123
6.788
6.784
6.771
6.767
6.670
6.664
6.653
6.647
6.576
6.571
6.366
6.362
2.707
2.704
2.696
2.493
0.970
H_2O
DMSO
(^{1}H, quintuplet)
14j
0.99
1.00
2.04
3.01
10.0
5.0
ppm (t1)

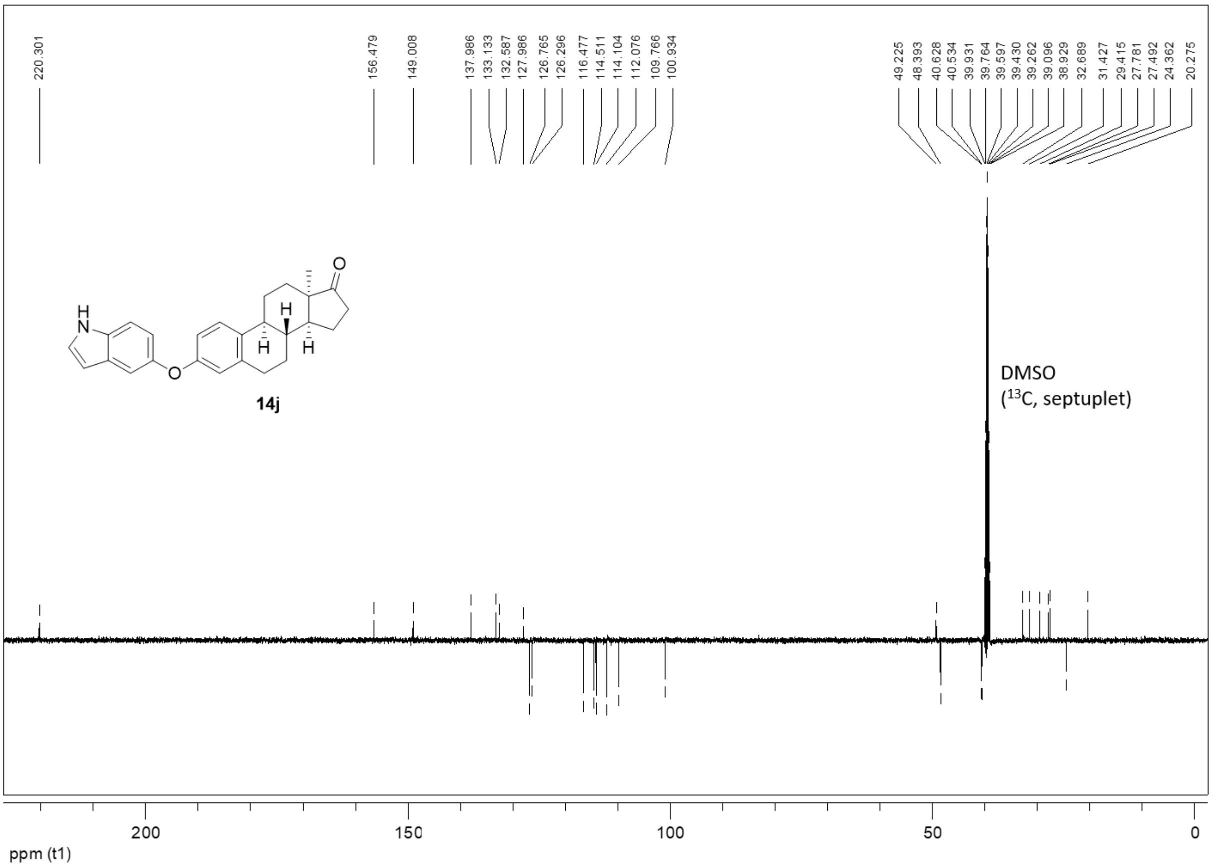

220.301
156.479
149.008
137.986
133.133
132.587
127.986
126.765
126.296
116.477
114.511
114.104
112.076
109.766
100.934
49.225
48.393
40.628
40.534
39.931
39.764
39.597
39.430
39.262
39.096
38.929
32.689
31.427
29.415
27.781
27.492
24.362
20.275
DMSO
(^{13}C, septuplet)
14j
200
150
100
50
0
ppm (t1)

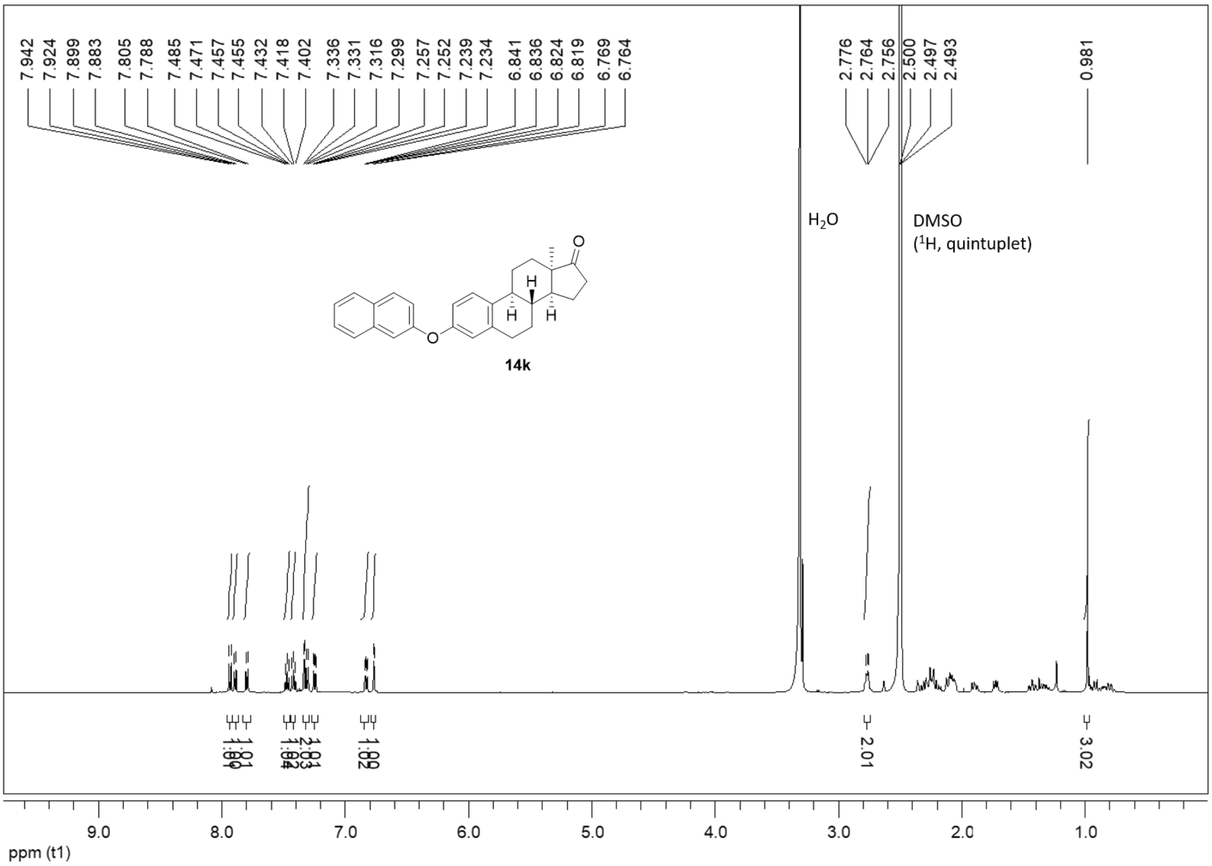
7.942 7.924 7.899 7.883 7.805 7.788 7.485 7.471 7.457 7.455 7.432 7.418 7.402 7.336 7.331 7.316 7.299 7.257 7.252 7.239 7.234 6.841 6.836 6.824 6.819 6.769 6.764
2.776 2.764 2.756 2.500 2.497 2.493
0.981
H_2O
DMSO
(1H, quintuplet)
14k
2.01
3.02
9.0 8.0 7.0 6.0 5.0 4.0 3.0 2.0 1.0
ppm (t1)

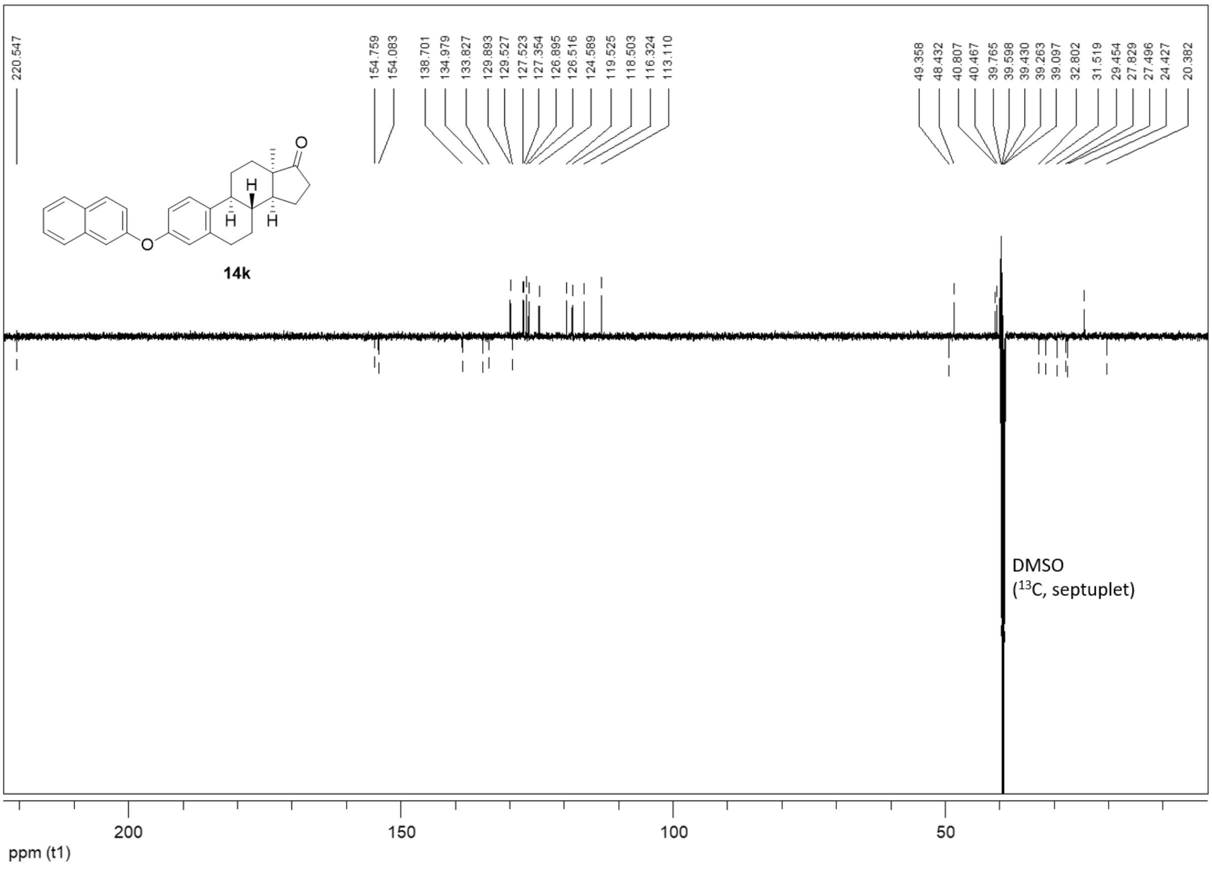
220.547
154.759 154.083
138.701 134.979 133.827 129.893 129.527 127.523 127.354 126.895 126.516 124.589 119.525 118.503 116.324 113.110
49.358 48.432 40.807 40.467 39.765 39.598 39.430 39.263 39.097 32.802 31.519 29.454 27.829 27.496 24.427 20.382
14k
DMSO
(13C, septuplet)
200 150 100 50
ppm (t1)

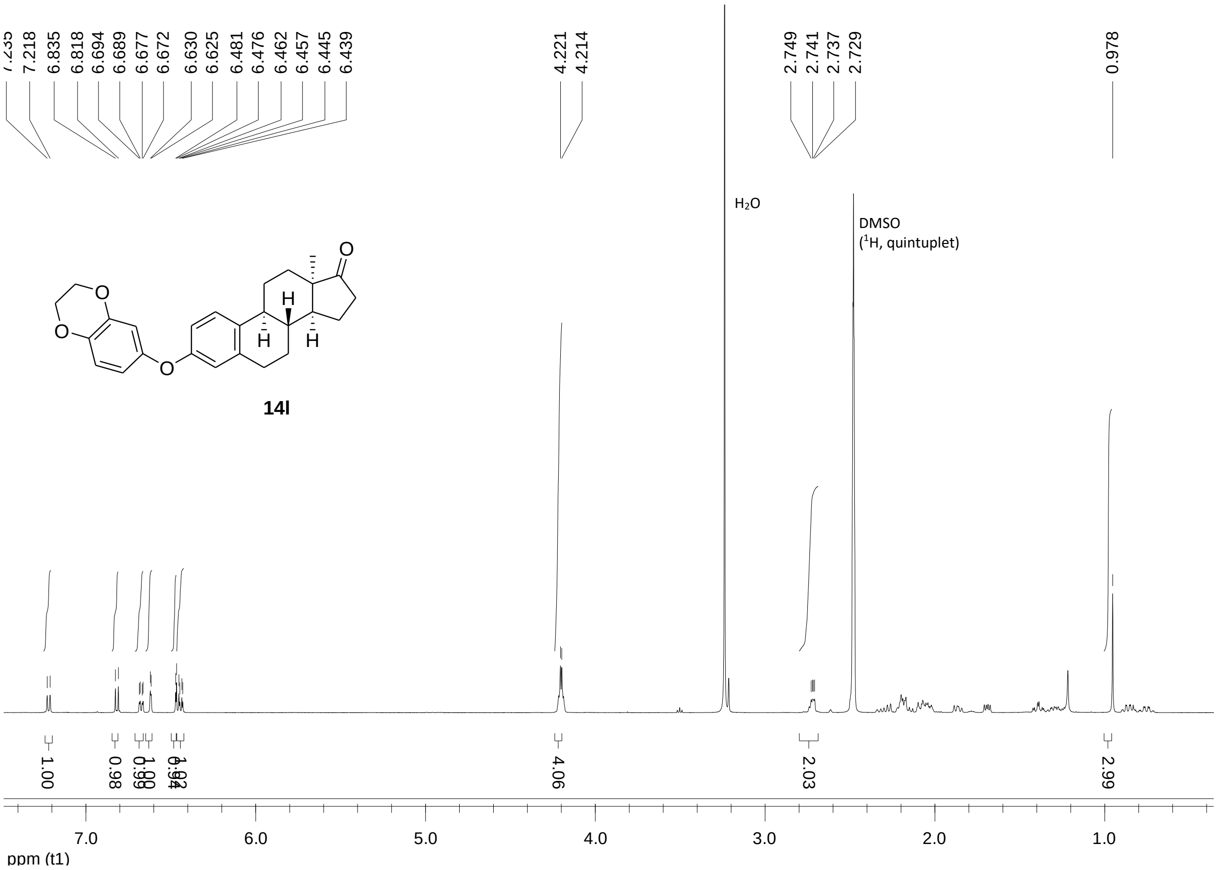

7.235
7.218
6.835
6.818
6.694
6.689
6.677
6.672
6.630
6.625
6.481
6.476
6.462
6.457
6.445
6.439
4.221
4.214
2.749
2.741
2.737
2.729
0.978
H_2O
DMSO
(1H, quintuplet)
14l
1.00
0.98
0.99
1.00
1.04
1.02
4.06
2.03
2.99
7.0
6.0
5.0
4.0
3.0
2.0
1.0
ppm (t1)

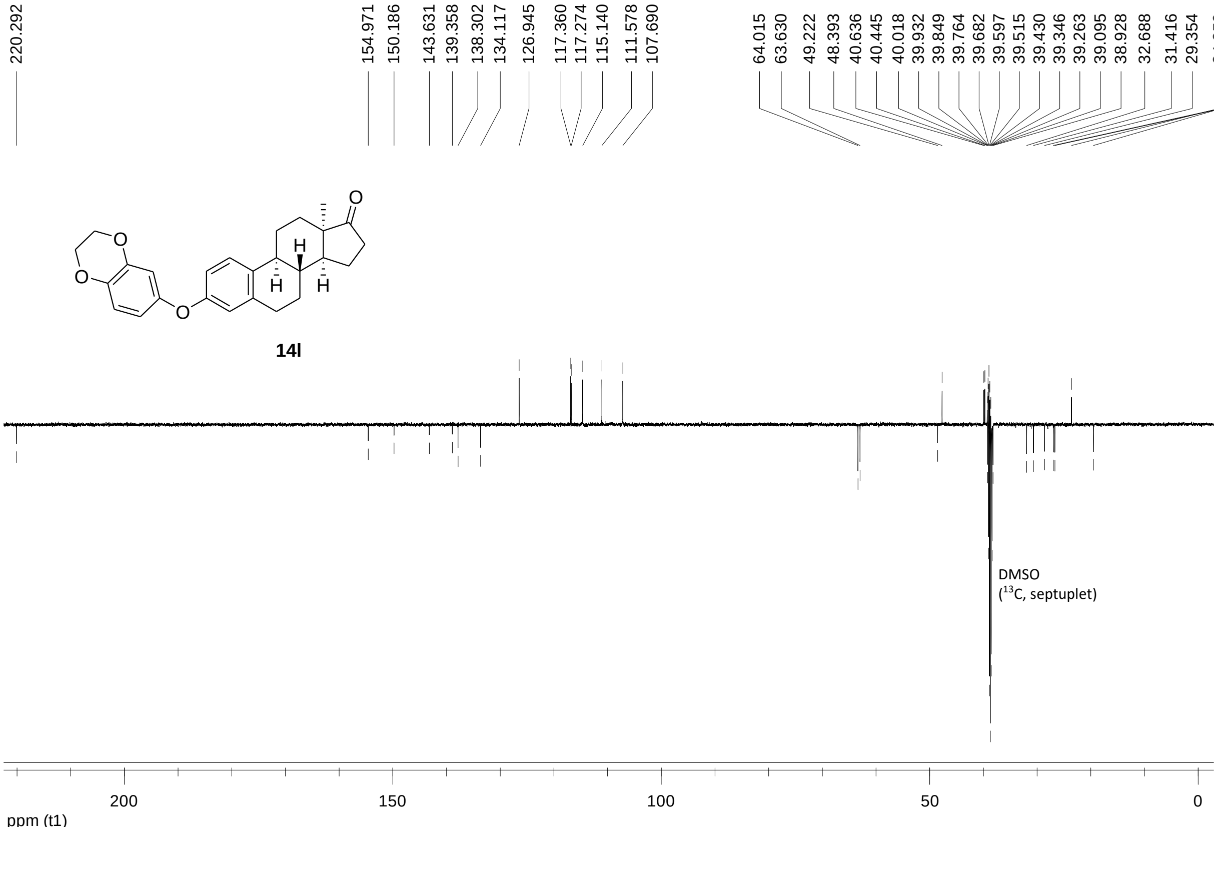

220.292
154.971
150.186
143.631
139.358
138.302
134.117
126.945
117.360
117.274
115.140
111.578
107.690
64.015
63.630
49.222
48.393
40.636
40.445
40.018
39.932
39.849
39.764
39.682
39.597
39.515
39.430
39.346
39.263
39.095
38.928
32.688
31.416
29.354
14l
DMSO
(13C, septuplet)
200
150
100
50
0
ppm (t1)
